# Dapagliflozin attenuates diabetic renal fibrosis by inhibiting macrophage-myofibroblast transition *via* the TGF-β1-Smad3/7 pathway

**DOI:** 10.7717/peerj.21321

**Published:** 2026-06-03

**Authors:** Xiaoyun Li, Guoxiang Yao, Honggang Wang, Tingting Zhao, Yujiao Sun, Qi Gao, Zhuo Li, Bing Chen

**Affiliations:** 1 Department of Nephrology, Shandong Provincial Hospital Affiliated to Shandong First Medical University, Jinan, Shandong, China; 2 Department of Nephrology, The Second Qilu Hospital of Shandong University, Jinan, Shandong, China; 3 Department of Respiratory and Critical Care Medicine, Shandong Public Health Clinical Center, Shandong University, Jinan, Shandong, China

**Keywords:** Diabetic kidney disease, Dapagliflozin, Renal fibrosis, Macrophage-myofibroblast transition (MMT), TGF-β1/Smad

## Abstract

**Objective:**

Renal fibrosis plays a pivotal role in the progression of renal dysfunction in diabetic kidney disease (DKD), with the macrophage-to-myofibroblast transition (MMT) serving as a central mechanism driving the advancement of renal fibrosis to end-stage renal disease (ESRD) in various chronic kidney conditions. Although the sodium-glucose cotransporter 2 (SGLT2) inhibitor dapagliflozin (DAPA) slows renal function decline in DKD, its antifibrotic mechanisms remain unclear. This study aimed to elucidate whether DAPA ameliorates renal fibrosis by suppressing MMT, and the underlying molecular mechanisms.

**Methods:**

We established a DKD model in male C57BL/6J mice by inducing them with a high-fat diet (HFD) followed by streptozotocin (STZ) injection. The effects of DAPA on renal function parameters (serum creatinine (Scr), urinary albumin-to-creatinine ratio (UACR)), and renal pathological injury, fibrosis markers (α-smooth muscle actin (α-SMA), type I collagen (Col-I)) were comprehensively evaluated. Immunofluorescence (IF) and Western blot were employed to analyze MMT progression (F4/80^+^/α-SMA^+^ double-positive cells) and TGF-β1-Smad3/7 pathway activity.

**Results:**

DAPA significantly reduced blood glucose levels, mitigated weight loss, and effectively inhibited type 2 diabetes-induced increase in Scr (43.78 ± 3.84 *vs.* 33.93 ± 6.77 µmol/L, *P* < 0.05) and UACR (89.17 ± 16.33 *vs.* 52.51 ± 10.51 mg/g, *P* < 0.05) in DKD mice. DAPA significantly attenuated glomerular hypertrophy, mesangial hyperplasia, and vacuolar degeneration of the tubular epithelium in DKD mice, while concurrently reducing glomerular and tubular injury scores, as well as the renal interstitial fibrosis area (*P* < 0.05). IF revealed increased MMT within the renal tubulointerstitium in DKD mice, accompanied by elevated deposition of α-SMA and Col-I. DAPA treatment markedly reduced F4/80^+^ macrophage infiltration and F4/80^+^/α-SMA^+^ and F4/80^+^/Col-I^+^ double-positive MMT cells, with decreased α-SMA and Col-I expression (*P* < 0.05). Additionally, TGF-β1 and phosphorylated Smad3 (p-Smad3) expression were significantly upregulated in DKD mice, whereas Smad7 was downregulated (*P* < 0.05). DAPA treatment significantly reduced TGF-β1 and p-Smad3 levels and restored Smad7 expression, rebalancing the Smad3/Smad7 axis (*P* < 0.05). Therefore, we propose that DAPA likely alleviates renal fibrosis in DKD by modulating renal TGF-β1 activity and restoring the Smad3/Smad7 balance, thereby suppressing MMT.

**Conclusion:**

This study is the first to reveal that DAPA is highly likely to exert anti-fibrotic effects through a novel mechanism involving targeted modulation of the TGF-β1-Smad3/7 axis to inhibit MMT, thereby laying a theoretical foundation and identifying a potential therapeutic target for precision treatment of DKD.

## Introduction

Chronic kidney disease (CKD) affects approximately 788 million adults worldwide (14.2% age-standardised prevalence) and ranks as the ninth leading cause of death globally, responsible for 1.48 million deaths annually (Collaborators, 2025). Diabetic kidney disease (DKD), a common microvascular complication of type 2 diabetes mellitus (T2DM), is characterized by persistent proteinuria and a progressive decline in the estimated glomerular filtration rate (eGFR), ultimately progressing to end-stage renal disease (ESRD) (Li et al., 2025a). The primary pathological mechanisms include hyperglycemia-induced glomerulosclerosis and tubulointerstitial fibrosis (Luo et al., 2025a). The current therapeutic strategies for DKD focus on the management of conventional risk factors—specifically, rigorous glycemic control, maintenance of blood pressure within target ranges, and renin-angiotensin system(RAS) inhibition (Wei et al., 2025). Renal fibrosis represents a critical pathological alteration driving DKD progression and serves as a common pathway for all CKD advancing to ESRD (Hong et al., 2025; Li et al., 2025b). DKD patients have substantial unmet medical needs. However, conventional agents such as angiotensin-converting enzyme inhibitors (ACEIs) and angiotensin receptor blockers (ARBs) exhibit limited efficacy in advanced nephropathy. Thus, novel renoprotective drugs targeting multiple pathological mechanisms, including glomerulosclerosis and interstitial fibrosis, are urgently required.

Dapagliflozin (DAPA), a novel sodium-glucose cotransporter 2 (SGLT2) inhibitor, reduces renal glucose reabsorption by up to 90% in the proximal tubules, thereby promoting urinary glucose excretion and lowering blood glucose levels, while demonstrating significant renoprotective effects (Liu et al., 2025). Clinical studies confirm that DAPA delays eGFR decline and mitigates renal fibrosis (Poursharif, Hamza & Braam, 2022), and animal studies further demonstrate that DAPA alleviates diabetic renal injury by inhibiting the TGF-β1/Smad signaling pathway (Xue et al., 2023). However, the precise mechanisms underlying DAPA’s therapeutic effects in DKD remain unclear.

In DKD, hyperglycemia-induced metabolic disturbances trigger chronic renal inflammation, where macrophages play dual roles in disease progression: promoting tissue repair through growth factor release while exacerbating inflammatory responses and driving fibrosis *via* pro-inflammatory mediators (*e.g.*, cytokines, TGF-β1) (Lin et al., 2023; Mitrofanova et al., 2022; Tang, Nikolic-Paterson & Lan, 2019). The pivotal pathological event in diabetic renal fibrosis involves the aberrant activation of myofibroblasts and their mediation of excessive extracellular matrix (ECM) deposition (Ma et al., 2025). Macrophage-myofibroblast transformation (MMT), a newly identified phenomenon driven by TGF-β1 signaling, represents a direct mechanism by which macrophages promote myofibroblast generation under renal inflammatory conditions (Tang, Nikolic-Paterson & Lan, 2019; Torres et al., 2020). Studies have demonstrated that in models of chronic renal allograft rejection and unilateral ureteral obstruction (UUO), bone marrow-derived macrophages differentiate into interstitial myofibroblasts, and the abundance of these cells is positively correlated with the extent of fibrosis (Chen et al., 2022a; Yao et al., 2025). In clinical specimens of fibrotic renal tissues, CD68/α-smooth muscle actin (α-SMA) double-positive cells were detected, and their quantity showed a positive correlation with both the overall myofibroblast population and the degree of renal functional impairment (Meng et al., 2016). Collectively, preclinical and clinical evidence has established MMT as a conserved driver of renal fibrogenesis; however, its specific role and molecular mechanisms in DKD-associated renal fibrosis remain elusive.

The TGF-β1/Smad3 signaling axis is a key regulator initiating MMT during renal fibrogenesis. Activation of the TGF-β1/Smad3 pathway induces macrophages to express α-SMA and type I collagen (Col-I), generating intermediate cells (MMT cells) co-expressing macrophage markers (F4/80, CD68) and myofibroblast markers (α-SMA). These cells directly promote excessive ECM deposition in the renal interstitium (Xia et al., 2025; Yang et al., 2025). This process highlights how macrophages translate inflammatory signals into fibrotic progression *via* a phenotypic transition, thereby providing a theoretical basis for targeting MMT to combat diabetic renal fibrosis. Beyond driving MMT, TGF-β1/Smad signaling critically modulates broader fibrotic pathways. As a well-established profibrotic mediator, TGF-β1 is highly expressed in multiple animal models of renal fibrosis (Dou et al., 2020; Ghafouri-Fard et al., 2024; Zhou et al., 2020). It modulates ECM production and degradation by activating downstream effectors (Yan et al., 2026). Smad3 is robustly activated. Smad7, a negative feedback regulator of TGF-β1/Smad signaling, inhibits Smad3 recruitment and phosphorylation (An et al., 2025; Wang et al., 2025b). Overexpression of Smad7 suppresses renal fibrosis and inflammation by blocking both TGF-β/Smad signaling pathways. Conversely, Smad7 deficiency exacerbates renal fibrosis and the inflammatory responses. Collectively, these findings indicated that the imbalance between Smad3 hyperactivation and Smad7 downregulation represents a critical pathogenic mechanism underlying renal fibrosis (He et al., 2024).

Building on this evidence, we hypothesize that DAPA’s renoprotective effects may involve modulation of the TGF-β1/Smad3-MMT network. To test this hypothesis, we employed a high-fat diet (HFD) combined with streptozotocin (STZ)-induced DKD mouse model to systematically evaluate DAPA’s efficacy in ameliorating renal fibrosis, with a focus on its regulatory roles in the TGF-β1-Smad3/7 signaling pathway and MMT. This study aimed to elucidate the novel antifibrotic mechanisms of DAPA and identify potential therapeutic targets for precise intervention in diabetic renal interstitial fibrosis.

## Materials & Methods

### Experimental animals and materials

Eighteen 8-week-old male C57BL/6J mice (weight 26.70 ± 1.79 g) were purchased from Jinan Pengyue Laboratory Animal Breeding Co., Ltd. (Jinan, China) and housed in the specific pathogen-free (SPF) facility of Shandong First Medical University Affiliated Provincial Hospital. The animals were maintained under controlled conditions (temperature, 22 ± 2 °C; humidity, 50% ± 10%; 12/12-hour light/dark cycle) with free access to food and water. Animals were housed at a density of no more than six animals per cage, providing a minimum floor area of 0.016 m^2^ per mouse to comply with welfare standards. For environmental enrichment, each cage was supplied with sterile bedding and a wooden chewing block. Per the ARRIVE guidelines and the European Union directive 2010/63/EU for animal experiments and in line with the National Institutes of Health guide for the care and use of Laboratory animals (NIH Publications 8023 revised 1978), the protocol of this study was approved by the Scientific Research Ethics Committee of the Second Hospital of Shandong University (Approval No. KYLL2024779; the institution is now renamed as “The Second Qilu Hospital of Shandong University”). STZ (S8050, Solarbio, Beijing, China) and DAPA (HY-10450; MedChemExpress Co., Ltd., Monmouth Junction, NJ, USA) were used.

### Animal grouping and model establishment

**Figure 1:**
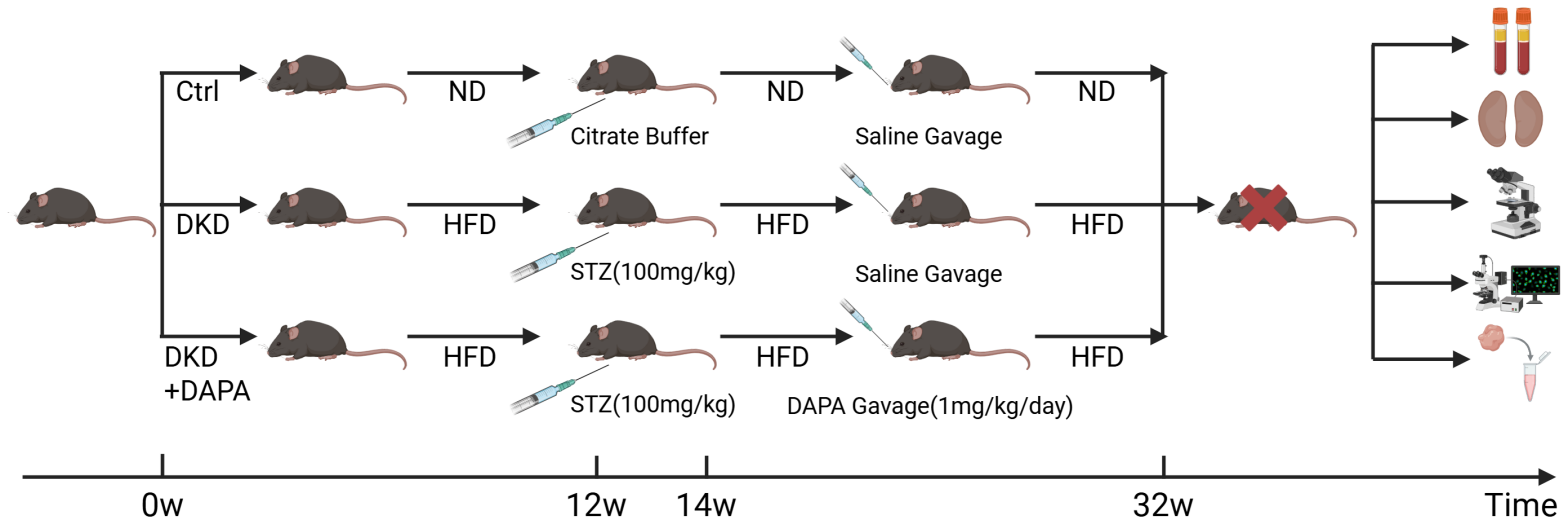
Schematic illustration of the experimental design and timeline. Eight-week-old male C57BL/6J mice were randomly divided into three groups: Ctrl (*n* = 6), fed normal diet (ND) and received citrate buffer (pH 4.5) intraperitoneally at week 12 followed by saline gavage daily from week 14 to week 32; DKD (*n* = 6), fed HFD (60% fat calories) for 12 weeks, received STZ (100 mg/kg) intraperitoneally at week 12, and continued HFD with saline gavage from week 14 to week 32; DKD+DAPA (*n* = 6), same DKD induction protocol with dapagliflozin (1 mg/kg/day) administered *via* oral gavage from week 14 to week 32. All mice were sacrificed at week 32 for sample collection. Created at BioRender.com.

After a 1-week acclimatization period, the mice were randomly divided into three groups: control (Ctrl, *n* = 6), DKD (*n* = 6), and DAPA treatment (DAPA, *n* = 6). The randomisation sequence was generated using the random number generation function in Microsoft Excel. After the random assignment, the mice were group-housed according to their designated groups for the duration of the study. The DKD and DAPA groups were fed a HFD (60% fat calories) for 12 weeks. After a 12-hour fast, the mice received an intraperitoneal injection of STZ (100 mg/kg, dissolved in citrate buffer, pH 4.5), whereas the control group received an equivalent volume of buffer. One week later, random blood glucose (RBG) levels were measured *via* tail vein sampling; mice with levels ≥16.7 mmol/L were considered successful T2DM models. Non-qualifying mice received an additional STZ injection (100 mg/kg) after a 12-hour fast. The Ctrl group received standard chow and saline gavage; the DKD group continued HFD feeding with saline gavage; the DAPA group received HFD and DAPA gavage (1 mg/kg/day, 0.2 mg/mL, for 4 months). Body weight was monitored biweekly, blood glucose was measured monthly, and 24-hour urine samples were collected using metabolic cages (Fig. 1).

Humane endpoints were defined according to previously established criteria (Zhang et al., 2025). No animals in this study met these criteria; all subjects maintained stable health parameters throughout the experimental timeline. Consistent with the terminal nature of the study design, no animals were retained beyond the experimental period. The sample size for each experimental group was six for all outcome measures. Of these, three randomly selected samples were subjected to Western blot analysis.

### Blood biochemistry and urinary protein analysis

After four months, the mice were euthanized by exposure to a 5% concentration of isoflurane for 2–3 min until respiratory arrest occurred, and death was confirmed by the absence of a pedal reflex, and blood and urine samples were collected for biochemical analysis. Whole blood was obtained *via* retro-orbital bleeding and centrifuged (12,000 rpm, 15 min, 4 °C) to isolate the serum. Serum creatinine (Scr) and urine creatinine (Ucr) levels were measured using commercial kits (C011-2-1, njjcbio, Nanjing, China). RBG levels were assessed using an AccuChek glucometer (1620368; Roche Diagnostics GmbH, Mannheim, Germany). Urinary protein was quantified *via* Coomassie Brilliant Blue (CBB) assay with a commercial kit (C035-2-1, njjcbio, Nanjing, China). Urine samples were reacted with CBB reagent, where NH_3_^+^ group binding triggered a colorimetric shift from brown to blue, and absorbance at 595 nm was measured.

### Histopathological analysis

As previously described (Dusabimana et al., 2020), renal tissues were fixed in 4% paraformaldehyde, paraffin-embedded, and sectioned (4 µm). Sections were deparaffinized, rehydrated, and stained with hematoxylin and eosin (H&E), periodic acid-Schiff (PAS), and Masson’s trichrome, followed by dehydration, clearing, mounting, and digital slide scanning using a TissueFAXS Plus ST whole-slide scanner (AP184121; TissueGnostics, Vienna, Austria).

Two blinded investigators independently performed quantitative analyses: 1. Glomerular Injury Score: Score based on the percentage of damaged glomerular area (0: no damage; 1: <25%; 2: 25–50%; 3: 50–75%; 4: >75%), according to Bowman’s space narrowing, mesangial matrix expansion, basement membrane thickening, and capillary tuft collapse (Dusabimana et al., 2020).

2. Tubular Injury Score: Graded by the proportion of tubular dilation, vacuolization, or brush border loss (0:0%; 1: ≤10%; 2:11–25%; 3:26–45%; 4:46–75%; 5: >75%) (Dusabimana et al., 2020).

3. Interstitial Fibrosis: Masson-positive areas (collagen deposition) were quantified using the QuPath software (×200 magnification, excluding glomerular and vascular regions).

Renal tubular injury was assessed by HE staining, glomerular damage was evaluated using PAS staining, and renal interstitial fibrosis was examined by Masson’s trichrome staining. Five randomly selected fields per section were analyzed.

### Immunohistochemistry and Immunofluorescence staining

After deparaffinization, rehydration, and heat-induced antigen retrieval (citrate buffer, pH 6.0, 98 °C, 20 min) of paraffin-embedded renal tissue sections, endogenous peroxidase blocking (3% H_2_O_2_), and blocking with 5% BSA. Primary antibodies were incubated overnight at 4 °C: TGF-β1 (A16640, 1:200, Abclonal, Wuhan, China), p-Smad3 (AP0727, 1:200, Abclonal, Wuhan, China), Smad7 (A16396, 1:200, Abclonal, Wuhan, China), α-SMA (A17910, 1:200, Abclonal, Wuhan, China), and Col-I (14695-1-AP, 1:1000, Proteintech, Wuhan, China). Following rinsing with PBS, the sections were incubated for one hour at room temperature with secondary antibodies conjugated to horseradish peroxidase.

For IF staining, F4/80 (A18637, 1:200, Abclonal, Wuhan, China) was labeled with PE (bs-0296G-PE, 1:500, BIOSS, Beijing, China), while α-SMA and Col-I were labeled with FITC (bs-0295G-FITC, 1:500, BIOSS, Beijing, China). Nuclei were counterstained with DAPI (C02-04002, Bioss, Beijing, China). Fluorescent images (×200 magnification, five fields per section) were captured using an Olympus BX63 microscope (Olympus Corporation, Tokyo, Japan). Protein expression levels were quantified by calculating the mean optical density (MOD) and average fluorenscence signal using the ImageJ software. MOD was calculated as the integrated optical density (IOD) divided by the region of interest (ROI) area (MOD = IOD/Area). Relative fluorescence intensity was normalized to that of the background signal.

### Western blot analysis

Renal tissues were homogenized in RIPA buffer (R0010, Solarbio, Beijing, China) containing 1% protease inhibitor (P1260, Solarbio, Beijing, China) (200 µL per 20 mg of tissue). Lysates were ultrasonicated on ice, centrifuged (12,000 ×g, 15 min, 4 °C), and supernatants were collected. Protein concentration was determined *via* BCA assay. The samples were denatured (95 °C, 5 min) using 5 × loading buffer (P0015, Beyotime, Shanghai, China). Proteins were separated on 10% SDS-PAGE gels (PG112, Epizyme, Shanghai, China) and transferred to PVDF membranes (BS-PVDF-45, 0.45 µm, Biosharp, Hefei, China). Membranes were blocked with 5% skim milk (1 h, room temperature), then incubated overnight at 4 °C with primary antibodies: TGF-β1 (A16640, 1:1000, Abclonal, Wuhan, China), p-Smad3 (AP0727, 1:1000, Abclonal, Wuhan, China), Smad3 (A22133, 1:2000, Abclonal, Wuhan, China), Smad7 (A16396, 1:2000, Abclonal, Wuhan, China), α-SMA (A17910, 1:2000, Abclonal, Wuhan, China), Col-I (14695-1-AP, 1:2000, Proteintech, Wuhan, China), and β-actin (db13986, 1:10,000, Diagbio, Hangzhou, China). After washing, the membranes were incubated with horseradish peroxidase-conjugated secondary antibodies (db10002, 1:20,000; Diagbio, Hangzhou, China) for 1 h at room temperature. Bands were visualized using ECL reagent (SQ201, Epizyme, Shanghai, China) and analyzed by ImageJ using grayscale densitometry (normalized to β-actin).

### Statistical analysis

Due to the nature of the treatment, the personnel responsible for the daily administration of DAPA or saline gavage (the investigators) were necessarily aware of the group allocation during the allocation and conduct of the experiment. However, to minimize bias, blinding was implemented during the outcome assessment and data analysis phases. The researchers performing the histological analyses (*e.g.*, Masson staining, IF staining), Western blot quantification, and measurements of biochemical indicators (*e.g.*, Scr, urine albumin) were unaware of the group identities. The data were analyzed using coded samples to ensure objectivity. Data were analyzed using GraphPad Prism version 10. All acquired data were retained for statistical analysis without exclusion. The normality and homogeneity of variance were also tested. Brown-Forsythe test was employed to verify homogeneity of variances. Normally distributed data are expressed as mean ± SD. Data normality was assessed using the Shapiro–Wilk test. Parametric tests were applied to normally distributed data with homogeneity of variance, one-way analysis of variance (ANOVA) followed by Tukey’s *post hoc* test was used for multiple group comparisons. For normally distributed data with unequal variances, Welch’s ANOVA followed by Games-Howell test was applied. When the normality assumption was violated, the non-parametric Kruskal-Wallis test was utilized, followed by Dunn’s test for multiple comparisons. *P* < 0.05 was considered statistically significant.

## Results

### DAPA reduces blood glucose and ameliorates renal function impairment in DKD

**Figure 2:**
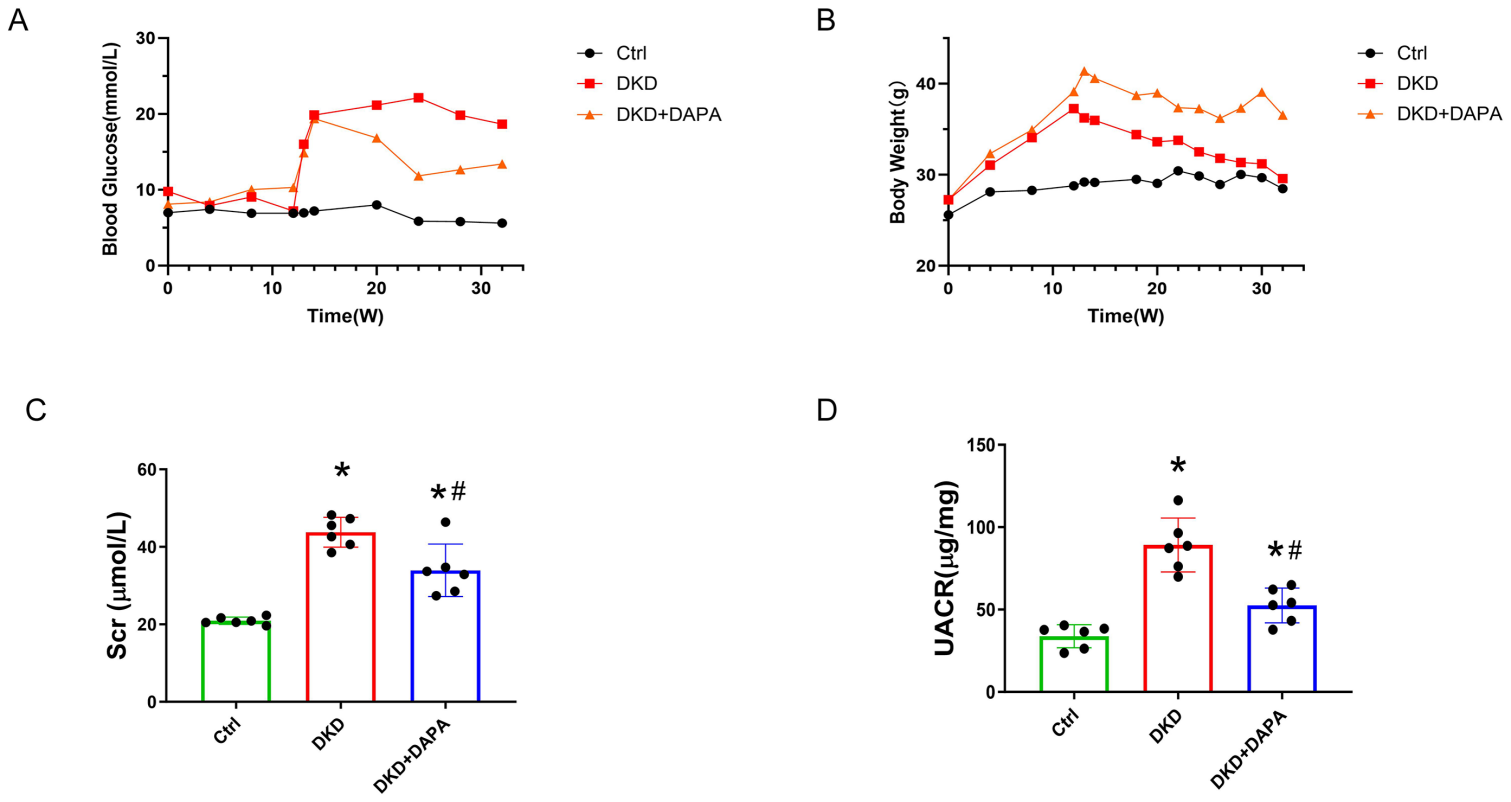
DAPA attenuates hyperglycemia, weight loss, and renal function impairment in HFD/STZ-induced DKD mice. (A) Random blood glucose levels measured throughout the 32-week experimental period. (B) Body weight changes monitored throughout the 32-week experimental period. (C) Scr levels at endpoint (week 32). Scr was significantly elevated in DKD mice, and DAPA treatment reduced Scr. (D) Measurement of UACR. UACR was increased in DKD mice compared to controls, and DAPA significantly decreased UACR. Data are presented as mean ± SD with individual data points (dots). *n* = 6 per group. *p < 0.05 *vs.* Ctrl group; #*p* < 0.05 *vs.* DKD group.

DAPA effectively reduced blood glucose levels and improved body weight loss. Compared to the Ctrl group, blood glucose levels were significantly elevated in the DKD group, whereas DAPA treatment markedly reduced blood glucose levels compared to those in the DKD group. Dapagliflozin showed significant glucose-lowering effects (Fig. 2A). Mice fed a HFD exhibited substantial weight gain. Following STZ induction, diabetic mice gradually lost weight, which was significantly attenuated by DAPA treatment (Fig. 2B).

DAPA effectively alleviated DKD-induced proteinuria and elevation of Scr. Scr levels were significantly elevated in the DKD group (43.78 ± 3.84 µmol/L *vs.* Ctrl: 20.92 ± 0.95 µmol/L, *P* < 0.0001), whereas DAPA treatment resulted in a 22.50% reduction in Scr compared to the DKD group (33.93 ± 6.77 µmol/L *vs.* DKD: 43.78 ± 3.84 µmol/L, *P* < 0.05) (Fig. 2C). Urinary albumin-to-creatinine ratio (UACR) in the DKD group was 2.64-fold higher than that in the Ctrl group (DKD: 89.17 ± 16.33 mg/g *vs.* Ctrl: 33.83 ± 7.04 mg/g, *P* < 0.0001). Following DAPA intervention, UACR decreased by 41.11% compared to the DKD group (52.51 ± 10.51 mg/g *vs.* DKD: 89.17 ± 16.33 mg/g, *P* < 0.05) (Fig. 2D).

### DAPA attenuates diabetic renal pathological injury and ameliorates glomerulosclerosis and interstitial fibrosis

**Figure 3:**
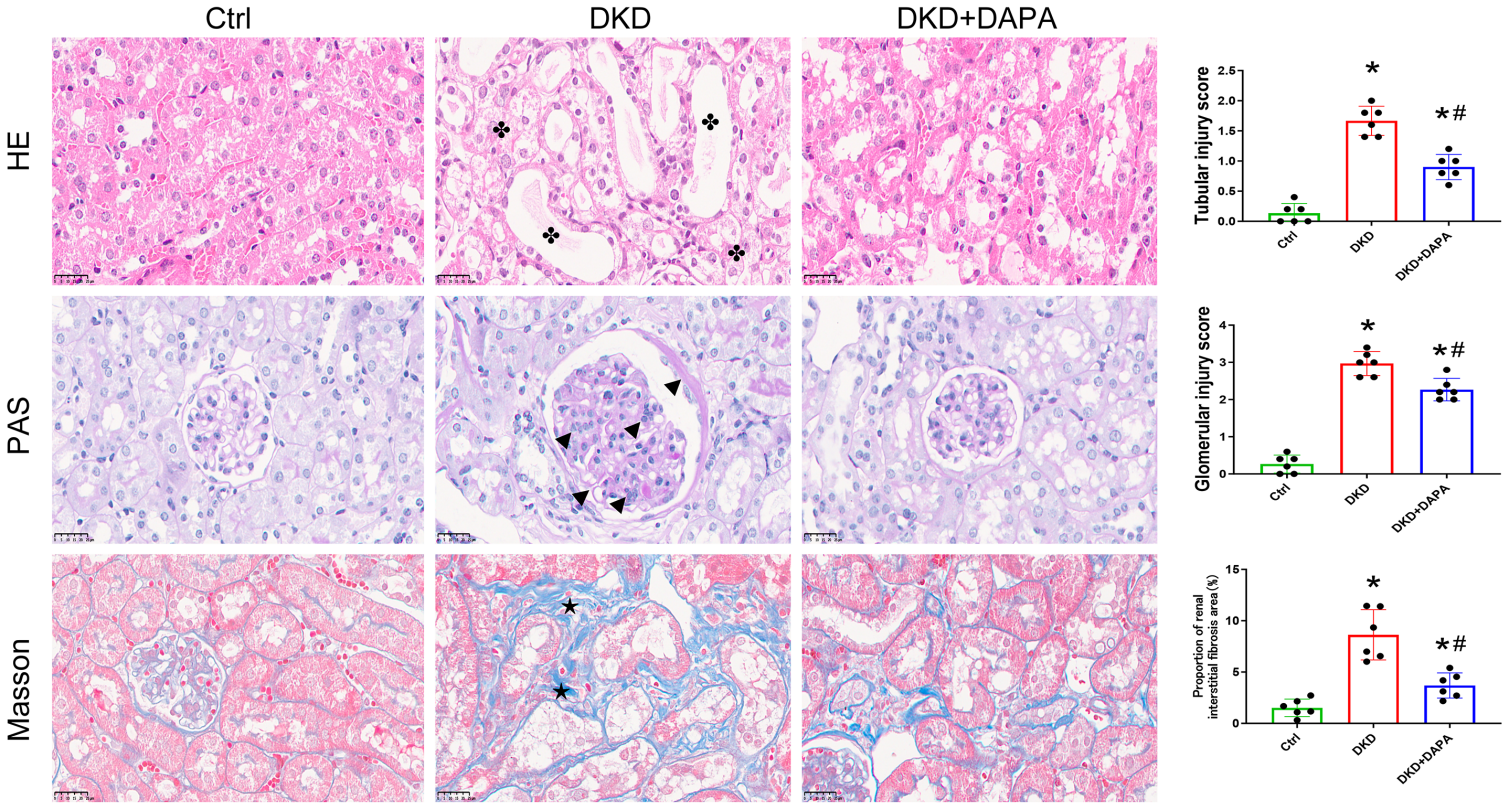
DAPA alleviates renal pathological injury and ameliorates glomerulosclerosis and interstitial fibrosis in diabetic nephropathy. H&E staining was performed to observe the histopathological morphology of renal tissues. DKD mice showed tubular dilation, brush border loss, and tubular epithelial vacuolization ( 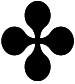). PAS staining was performed to assess glomerular injury (magenta-positive area). DKD mice exhibited mesangial matrix expansion, basement membrane thickening, and capillary tuft collapse (▴). Masson’s trichrome staining was performed to detect interstitial fibrosis (blue areas, ⋆). Marked collagen deposition in tubulointerstitium of DKD mice was significantly reduced by DAPA. Scale bar = 25 µm. *n* = 6 per group. Data are presented as mean ± SD with individual data points (dots). *p < 0.05 *vs.* Ctrl group; #*p* < 0.05 *vs.* DKD group.

Histological examination of renal tissues revealed that the DKD group displayed typical diabetic nephropathy pathologies, including segmental or global glomerular hypertrophy, marked mesangial cell proliferation, narrowed Bowman’s capsule spaces, proximal tubular epithelial cytoplasmic vacuolization, and focal brush border loss. The DAPA group showed a significant mitigation of glomerular hypertrophy, reduced mesangial hyperplasia, restored Bowman’s capsule spaces, and decreased tubular epithelial vacuolization (Fig. 3).

DAPA intervention significantly reduced both glomerular damage and tubular injury scores in DKD mice (*P* < 0.05). PAS staining demonstrated a uniformly distributed mesangial matrix in the Ctrl group, which was consistent with normal morphology. The DKD group exhibited mesangial area expansion, mesangial cell proliferation, homogeneous basement membrane thickening, and tubular epithelial atrophy/degeneration, leading to significantly elevated glomerular and tubular injury scores compared with the Ctrl group. DAPA treatment alleviated these symptoms (Fig. 3).

Furthermore, DAPA mitigated interstitial fibrosis. Masson’s trichrome staining revealed sparse collagen fibers (blue staining) in the renal interstitium of the Ctrl group. The DKD group showed marked collagen deposition in the tubulointerstitium, indicative of interstitial fibrosis, with a significantly increased fibrotic area compared to the Ctrl group (*P* < 0.05). DAPA treatment significantly reduced the fibrotic area (*P* < 0.05) (Fig. 3).

### DAPA attenuates macrophage infiltration and ECM (Col-I, α-SMA) deposition in diabetic kidneys

**Figure 4:**
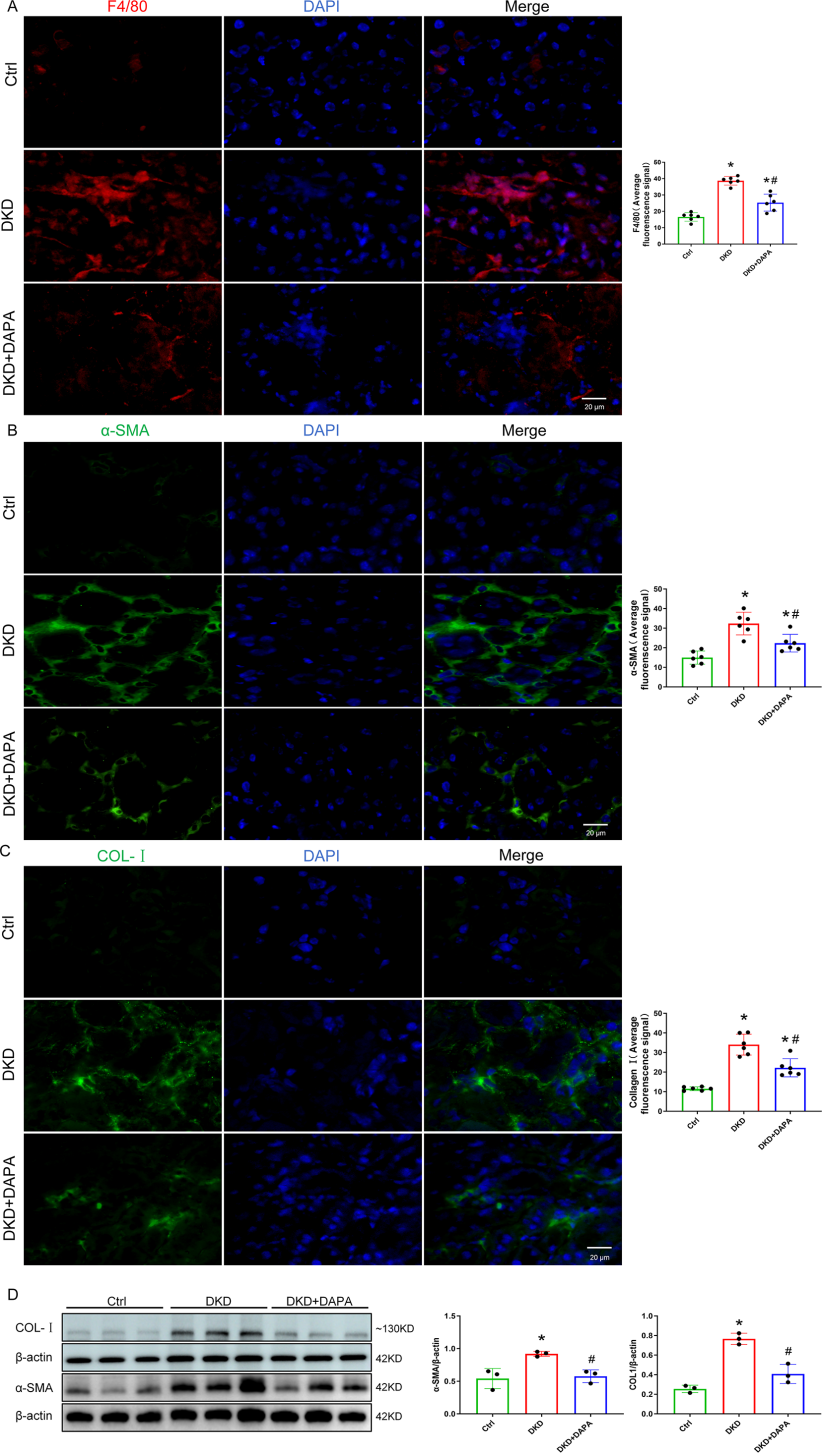
DAPA attenuates macrophage infiltration and ECM (Collagen I, α-SMA) deposition in diabetic kidneys. (A–C) Representative IF staining images showing F4/80 (macrophage marker, red), α-SMA (myofibroblast marker, green), and Col-I (green) in renal tubulointerstitium. DAPI (blue) indicates nuclei. Diabetic mice showed increased interstitial expression of F4/80^+^ macrophages, α-SMA^+^ myofibroblasts, and Col-I, which was reduced by DAPA treatment. Scale bar = 20 µm. (D) Western blot analysis of α-SMA and Col-I protein expression levels in renal tissues (normalized to β-actin). α-SMA and Col-I expression was increased in diabetic kidneys and decreased following DAPA treatment. Data are presented as mean ± SD with individual data points (dots). *n* = 6 per group for IF; *n* = 3 per group for Western blot. *p < 0.05 *vs.* Ctrl group; #*p* < 0.05 *vs.* DKD group.

Using IF staining, we observed a significantly elevated expression of the macrophage marker F4/80 in the renal tissues of DKD mice compared to that in the Ctrl group (*P* < 0.05), which was markedly reduced by DAPA treatment (*P* < 0.05) (Fig. 4A). Furthermore, fibrosis markers associated with MMT—Col-I and α-SMA—were also significantly upregulated in DKD mice (*P* < 0.05), and both synchronously decreased after DAPA intervention (*P* < 0.05) (Figs. 4B, 4C). Western blot analysis further confirmed that α-SMA and Col-I protein levels were significantly lower in DAPA-treated kidneys than in untreated DKD mice (*P* < 0.05) (Fig. 4D).

### DAPA exerts anti-fibrotic effects by suppressing the MMT process in renal tissues of DKD mice

**Figure 5:**
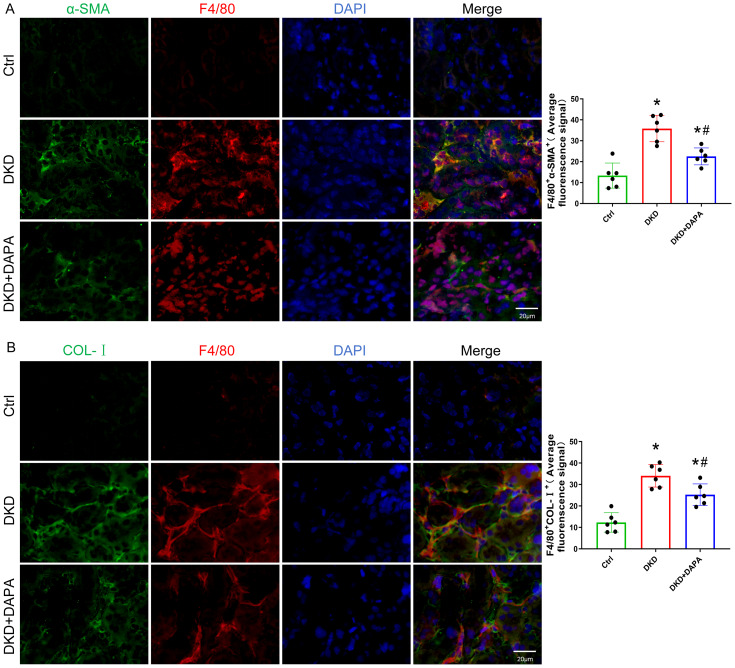
DAPA exerts anti-fibrotic effects by suppressing the MMT process in renal tissues of DKD mice. (A) IF co-staining of the macrophage marker F4/80 (red) and myofibroblast marker α-SMA (green) in the renal interstitium. Double-positive cells (F4/80^+^ /α-SMA^+^, MMT cells, yellow/orange) were observed in DKD mice, whereas their number was significantly reduced in the DAPA-treated group. (B) IF analysis revealed co-localization of COL-1 (green) with the macrophage marker F4/80 (red) in the renal interstitium. Co-localized signals (yellow/orange) were markedly increased in DKD mice and significantly decreased after DAPA treatment. Scale bar = 20 µm. Data are presented as mean ± SD with individual data points (dots). *n* = 6 per group. *p < 0.05 *vs.* Ctrl group; #*p* < 0.05 *vs.* DKD group.

Increasing evidence in recent years has demonstrated that bone marrow-derived macrophages (BMDMs) are a significant source of myofibroblasts. The presence of MMT in BMDMs represents a novel and critical mechanism that drives renal fibrosis. Using dual IF labeling, we detected a substantial infiltration of MMT cells (co-labeled cells) within the renal interstitium of DKD mice. DAPA treatment significantly attenuated this infiltration (Figs. 5A, 5B). These findings indicate that MMT contributes to renal fibrogenesis and that DAPA ameliorates renal fibrosis by suppressing MMT in DKD mice.

### DAPA suppresses TGF-β1 and p-Smad3 expression, rebalances Smad3/7 signaling, and attenuates renal fibrosis in DKD

**Figure 6:**
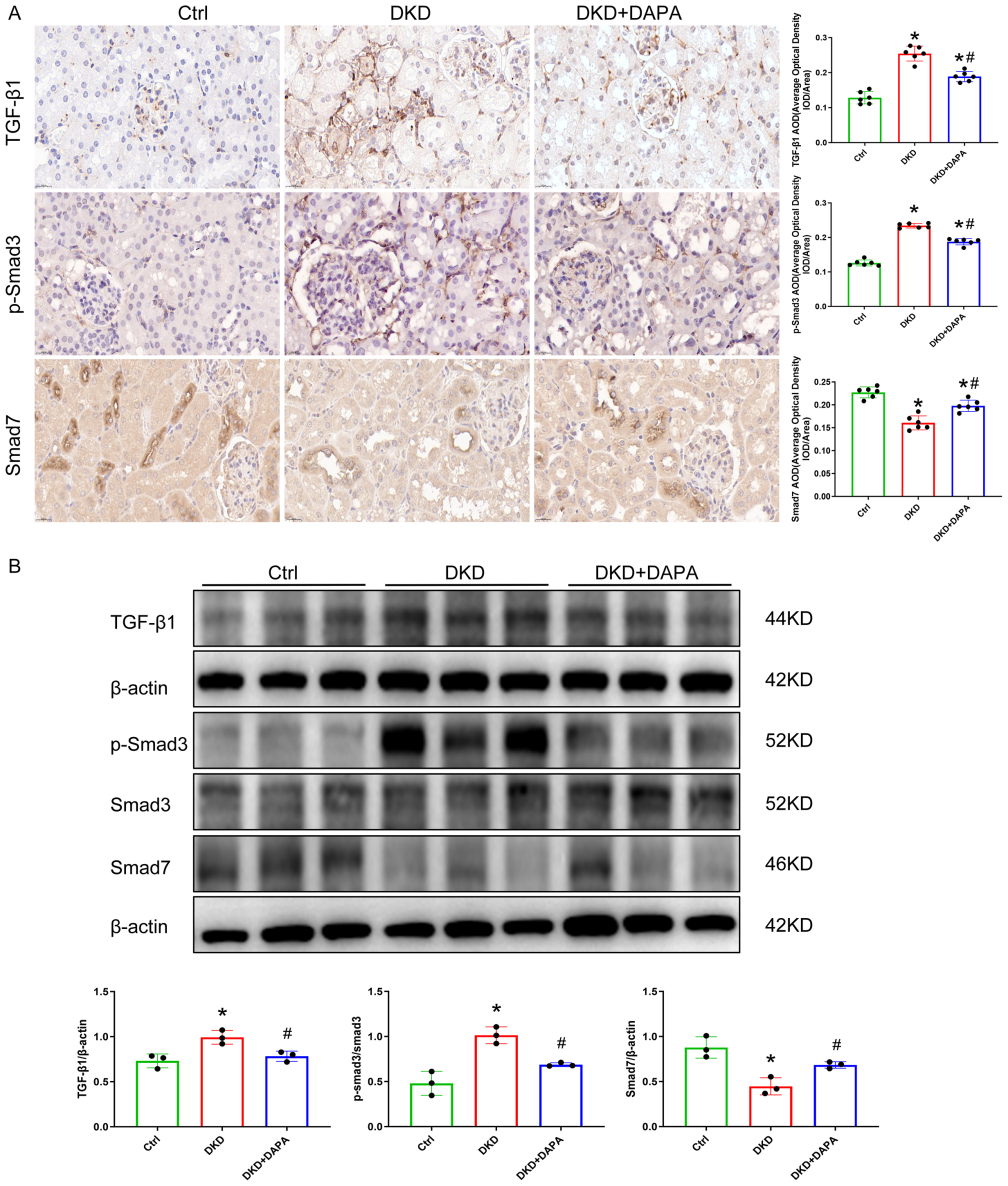
DAPA ameliorates DKD-induced renal fibrosis by inhibiting TGF-β1 and p-Smad3 expression. (A) Representative IHC staining images of TGF-β1, p-Smad3, and Smad7 in renal tissues. Diabetic mice showed increased TGF-β1/p-Smad3 and decreased Smad7, which was reversed by DAPA. Scale bar = 2 0 µm. (B) Western blot analysis of TGF-β1, p-Smad3, and Smad7 protein expression in renal tissues (normalized to β-actin). TGF-β1 and p-Smad3/Smad3 ratio were increased and Smad7 was decreased in diabetic mice, and DAPA restored the Smad3/Smad7 balance. Data are presented as mean ± SD with individual data points (dots). *n* = 6 per group for I HC; *n* = 3 per group for Western blot. *p < 0.05 *vs.* Ctrl group; #*p* < 0.05 *vs.* DKD group.

IHC analysis revealed expression of TGF-β1, p-Smad3, and Smad7 proteins in both glomeruli and renal tubules (Fig. 6A). The Ctrl group exhibited minimal deposition of TGF-β1 and p-Smad3 proteins in glomeruli and tubules, along with abundant Smad7 deposition. Compared to the Ctrl group, the DKD group showed significantly increased deposition and upregulated protein expression of TGF-β1 and p-Smad3 in glomeruli and tubules (*P* < 0.05), indicating overactivation of the TGF-β1/Smad3 pathway. Concurrently, Smad7 expression was markedly reduced (*P* < 0.05), leading to the loss of its inhibitory effect on Smad3 phosphorylation. DAPA treatment effectively reduced TGF-β1 and p-Smad3 deposition, significantly downregulated their protein expression (*P* < 0.05), and restored Smad7 activity in renal tissues (Fig. 6B).

## Discussion

DKD remains a major determinant of morbidity and mortality in diabetic patients (Rayego-Mateos et al., 2023). Although intensive glucose-lowering and antihypertensive therapies can retard DKD progression in some patients, most patients experience persistent disease advancement owing to “hyperglycemic memory” and associated epigenetic alterations. These patients ultimately require renal replacement therapy, which imposes a substantial economic burden on their families and society. Consequently, DKD represents a condition with profound unmet medical needs, underscoring the critical urgency of elucidating its underlying mechanisms and developing novel therapeutic strategies.

SGLT2 inhibitors are a novel class of agents that reduce renal glucose reabsorption and promote urinary glucose excretion, thereby improving glycemic control (Luo et al., 2025b). Poursharif, Hamza & Braam (2022) demonstrated that SGLT2 inhibitors preserved renal function by attenuating the decline in eGFR in both early- and late-stage DKD. DAPA, a clinically validated SGLT2 inhibitor (McEwan et al., 2022; Schechter et al., 2023; Zhang & Deng, 2025), effectively mitigates renal injury and suppresses fibrosis in DKD, making it a promising therapeutic option. However, the precise mechanisms underlying DAPA’s renoprotective effects of DAPA in DKD remain unclear and require further investigations.

In this study, we demonstrated that DAPA, a selective SGLT2 inhibitor, lowered blood glucose levels and proteinuria, attenuated body weight loss, and improved renal function (*e.g.*, reduced Scr and UACR levels) in DKD mice. Beyond improving renal function and metabolic outcomes, DAPA has been demonstrated to ameliorate DKD through multiple pathways, including attenuation of oxidative stress (Yaribeygi et al., 2019a; Yaribeygi et al., 2019b), suppression of inflammation (La Grotta et al., 2022), modulation of metabolism (Androutsakos et al., 2022), enhancement of mitochondrial function (Yaribeygi et al., 2023; Yaribeygi et al., 2022), and inhibition of fibrosis (Afsar & Afsar, 2023). Among these, suppression of renal fibrosis, particularly tubulointerstitial fibrosis, represents the core mechanism by which DAPA mitigates the pathological progression of DKD. Studies have indicated that DAPA effectively attenuates the development of renal fibrosis primarily by blocking the epithelial-mesenchymal transition (EMT) process, mitigating inflammatory responses, alleviating hypoxia and oxidative stress, and inhibiting the overactivation of the RAS (Afsar & Afsar, 2023; Xie et al., 2024). Our study provides the first evidence that DAPA inhibits MMT and suggests that this novel mechanism contributes to its antifibrotic effects.

The suppression of MMT may represent one of the mechanisms underlying the antifibrotic effects of DAPA in DKD. Our study revealed substantial infiltration of macrophages and MMT cells into the fibrotic kidneys of DKD mice, accompanied by pronounced renal fibrosis and functional decline, suggesting a biological process wherein macrophages acquire myofibroblast-like characteristics *via* phenotypic transition (*i.e.,* MMT) within injured renal tissues (Chen et al., 2022a; Wang et al., 2022). This phenomenon is consistent with previous observations of the MMT in cardiac, hepatic, and pulmonary fibrosis studies. Notably, MMT has been shown to directly contributes to approximately 35% of myofibroblasts in pulmonary fibrosis models, indicating its potential role as a shared mechanism in organ fibrosis and a critical driver of fibrotic progression (Little et al., 2020; Shen et al., 2025; Vierhout et al., 2021; Xia et al., 2023). In murine models of chronic renal transplant rejection, approximately 50% of interstitial myofibroblasts originate from bone marrow-derived macrophages, a proportion rising to 65% in UUO models, and their abundance is positively correlated with fibrosis severity (Chen et al., 2022a; Yang et al., 2023; Yao et al., 2025). Similarly, clinical samples of fibrotic renal tissues exhibit CD68^+^/α-SMA^+^ cells, the number of which significantly correlates with total myofibroblast counts, fibrosis extent, and renal dysfunction (Meng et al., 2016; Wei, Xu & Yan, 2022). Our findings demonstrate that DAPA significantly suppresses the upregulated expression of macrophage marker F4/80, myofibroblast marker α-SMA, and fibrosis-associated Col-I in DKD mice. IF co-staining further revealed co-localized expression of α-SMA/Col-I with macrophage marker F4/80 in the renal interstitium of DKD mice. DAPA treatment markedly reduced the number of cells co-expressing α-SMA/Col-I and F4/80, indicating its inhibition of macrophage-to-myofibroblast transition and consequent attenuation of renal fibrosis.

During the progression of DKD, persistent renal inflammation and fibrosis ultimately lead to renal injury (Speeckaert, Delrue & Speeckaert, 2026). Multiple signaling pathways contribute to the pathological processes of inflammation and renal fibrosis in DKD, among which the TGF-β1-mediated Smad signaling pathway is a hallmark pathway (Wang et al., 2025c). TGF-β1 is highly expressed in various renal resident cells and infiltrating inflammatory cells, regulating both Smad-dependent and Smad-independent signaling pathways (Aihara et al., 2010). Recent studies have further elucidated the critical role of TGF-β1/Smad signaling in diabetic renal fibrosis. Xue et al. (2023) demonstrated that DAPA alleviates renal fibrosis in T2DM rats by inhibiting the TGF-β1/Smad pathway. Furthermore, emerging evidence demonstrates that TGF-β1/Smad3 signaling drives renal fibrosis through multiple mechanisms, including activation of renal interstitial fibroblasts, promotion of tubular EMT, and induction of MMT (Hung et al., 2021). Notably, recent studies have identified novel regulatory nodes in this pathway, such as Piezo1-mediated mechanotransduction that modulates TGF-β1/Smad signaling in diabetic kidney fibrosis (Yan et al., 2025), and the potential of targeted TGF-β receptor-mimicking peptides to enhance inhibition of TGF-β1/Smad and p38 MAPK pathways for improved kidney fibrosis therapy (Wang et al., 2024). Consistent with these findings, DAPA has been shown to ameliorate fibrosis by suppressing the TGF-β1/Smad3 axis and restoring Smad3/Smad7 balance (Chen et al., 2022b; Pan et al., 2021; Wang et al., 2025a; Zhang et al., 2021b).

Smad7 serves as a critical negative regulator of the TGF-β/Smad signaling cascade, and an imbalance between Smad3 and Smad7 signaling may constitute a key mechanism underlying fibrogenesis (Gu et al., 2020; Lyu et al., 2023). Smad3-knockout mice or Smad3 inhibitors alleviate renal fibrosis and inflammation in diabetic mice, indicating the critical role of TGF-β1/Smad3 signaling in DKD pathogenesis (Wu et al., 2021; Xu et al., 2020). Conversely, Smad7, an inhibitory Smad, suppresses TGF-β1/Smad3 signaling by interacting with TGF-β1 receptors and acts as an antagonist in DKD, significantly attenuating microalbuminuria, TGF-β/Smad3-mediated renal fibrosis (*e.g.*, type I and IV collagen), and fibronectin accumulation in diabetic rats (Chen et al., 2011). Thus, inhibiting TGF-β1/Smad3 signaling represents a pivotal therapeutic strategy to mitigate renal inflammation and fibrosis in DKD (Gu et al., 2020; Xu et al., 2020). Combinatorial therapy with the Smad7 agonist asiatic acid and Smad3 inhibitor naringenin (AANG) effectively prevented T2DM and DKD in early prediabetic (but not in diabetic) db/db mice (Chung et al., 2022).

MMT is primarily regulated by TGF-β/Smad3 signaling, as genetic ablation of Smad3 in the bone marrow compartment of GFP+ chimeric mice prevented macrophage transition into MMT cells and attenuated renal fibrosis progression, while TGF-β1/Smad3 pathway activation induces macrophages to express α-SMA and type I collagen, generating MMT cells that directly promote ECM deposition in the renal interstitium (Wang et al., 2016). Multiple studies have demonstrated that the TGF-β1/Smad3 pathway critically coordinates macrophage infiltration, M2 polarization, and MMT during fibrogenesis (Tang et al., 2024; Tang et al., 2020; Yao et al., 2025). Our study found significantly upregulated TGF-β1 and p-Smad3 expression alongside downregulated Smad7 in DKD mice, indicating hyperglycemia-induced disruption of TGF-β1/Smad signaling that disrupts Smad3/Smad7 balance and drives renal fibrosis. DAPA treatment markedly reversed these alterations, demonstrating its dual modulation of the TGF-β1–Smad3/7 axis and inhibition of MMT. MMT occurs predominantly in M2 macrophages, which undergo transdifferentiation into myofibroblast-like cells and secrete ECM components to exacerbate fibrosis (Ban et al., 2024; Yao et al., 2025; Zhang, Li & Li, 2021a). However, phenotypic characterization of macrophage subsets was not performed in this study, precluding a direct assessment of whether myofibroblasts originate from the M2 macrophage transition. This is a limitation of the present study.

Our findings from this preclinical study have important implications for clinical practice. Recent landmark clinical trials, including DAPA-CKD (Heerspink et al., 2020) and EMPA-KIDNEY (Herrington et al., 2023), have demonstrated the renoprotective effects of SGLT2 inhibitors in patients with chronic kidney disease, independent of glycemic control. The DAPA-CKD trial specifically showed that DAPA reduced the risk of kidney failure and prolonged survival in patients with CKD, including those with diabetic nephropathy. Our mechanistic study provides a potential explanation for these clinical observations, suggesting that DAPA’s anti-fibrotic effects may be mediated through modulation of the TGF-β1-Smad3/7-MMT axis. However, whether MMT inhibition contributes to the renoprotective effects of SGLT2 inhibitors in human DKD remains to be validated in clinical studies. This represents another limitation of the present study. Future research should investigate the presence of MMT in kidney biopsies from DKD patients treated with SGLT2 inhibitors and explore the correlation between MMT markers and clinical outcomes.

**Figure 7:**
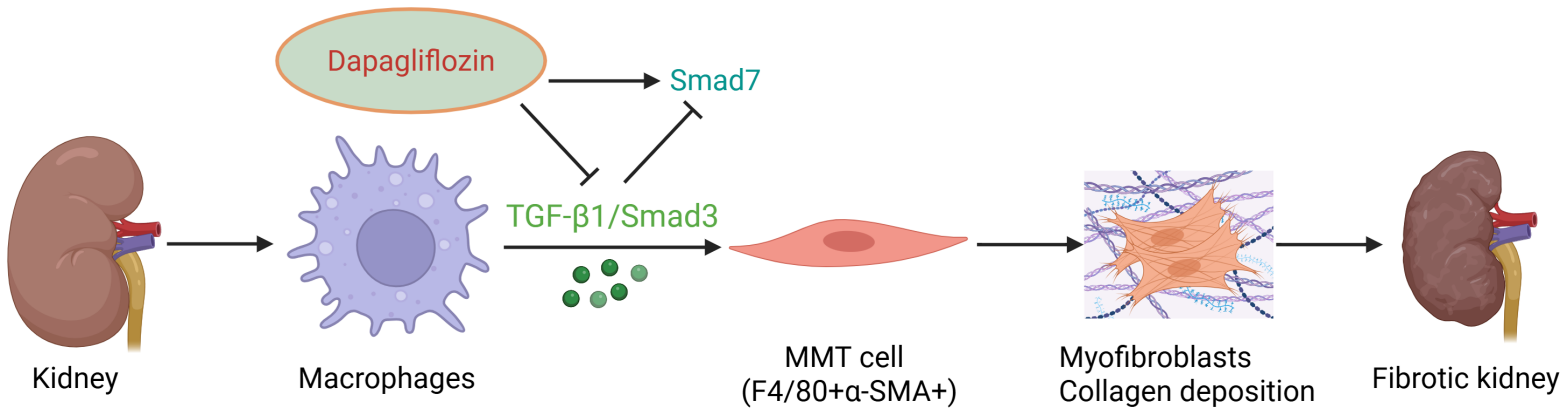
Mechanistic schematic illustrating DAPA-mediated attenuation of diabetic renal fibrosis through inhibition of MMT. In DKD, hyperglycemia induces renal inflammation and activation of the TGF-β1/Smad3 pathway, which drives macrophages (F4/80^+^) to undergo MMT, acquiring myofibroblast characteristics (α-SMA^+^) and producing Col-I, leading to tubulointerstitial fibrosis and progressive kidney dysfunction. DAPA inhibits TGF-β1/Smad3 pathway activity, upregulates Smad7 expression, restores Smad3/Smad7 homeostatic balance, and is highly likely to block the MMT process through this mechanism, thereby reducing α-SMA and Col-I deposition, and ultimately ameliorating diabetic renal fibrosis. Created at BioRender.com.

Our findings extend previous research by providing the first evidence that DAPA may ameliorate renal fibrosis by modulating the TGF-β1–Smad3/7 axis to suppress macrophage-driven MMT, thereby revealing a potential antifibrotic mechanism (Fig. 7).

## Conclusions

This study innovatively reveals that DAPA is highly likely to alleviate renal fibrosis in DKD through a novel molecular mechanism involving targeting the TGF-β1-Smad3/7 signaling axis to regulate the MMT process, ultimately mitigating fibrotic progression. This is the first study to demonstrate that DAPA reduces α-SMA^+^/Col-I^+^ myofibroblast accumulation by inhibiting MMT, thereby highlighting a novel therapeutic target for diabetic renal fibrosis. Unlike conventional antifibrotic agents, which primarily focus on direct fibroblast suppression, our study highlights the pivotal role of immune cell phenotypic transition in DKD pathogenesis. Mechanistically, DAPA suppresses TGF-β1/Smad3 pathway activity, upregulates Smad7 expression, and restores the Smad3/Smad7 dynamic equilibrium, achieving dual inhibition of pro-fibrotic signaling cascades. Importantly, this study not only confirms the glucose-independent renoprotective effects of SGLT2 inhibitors in DKD but also identifies the TGF-β1-Smad3/7-MMT axis as a precision therapeutic target through multidimensional molecular interaction analyses (IF co-localization coupled with spatial IHC quantification). These findings establish a theoretical foundation for the development of next-generation antifibrotic drugs targeting MMT.

##  Supplemental Information

10.7717/peerj.21321/supp-1 Supplemental Information 1 Raw data and ARRIVE Checklist

## Additional Information and Declarations

### Data Availability

The following information was supplied regarding data availability: The raw measurements are available in the Supplemental File.
