# Supplementary material for: Dapagliflozin attenuates diabetic renal fibrosis by inhibiting macrophage-myofibroblast transition via the TGF-β1-Smad3/7 pathway
Source: PeerJ. 2026 Jun 3;14:e21321. doi: 10.7717/peerj.21321 (PMC13242187; doi:10.7717/peerj.21321)
Supplement: Supplemental Information 1 [file peerj-14-21321-s001.zip › Supplementary Document/Western Blot raw data/Western Blot Raw Data Instructions.pdf]

Western Blot Raw Data Instructions

α -SMA

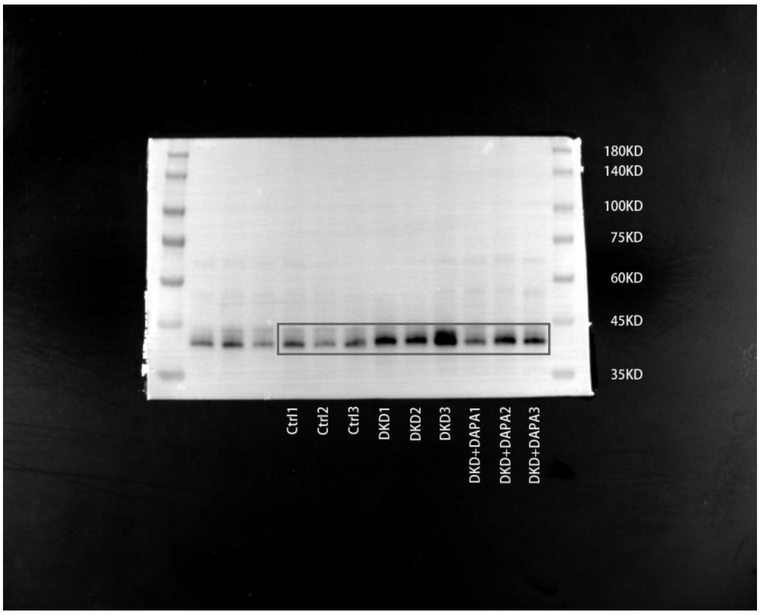

α -SMA β -actin

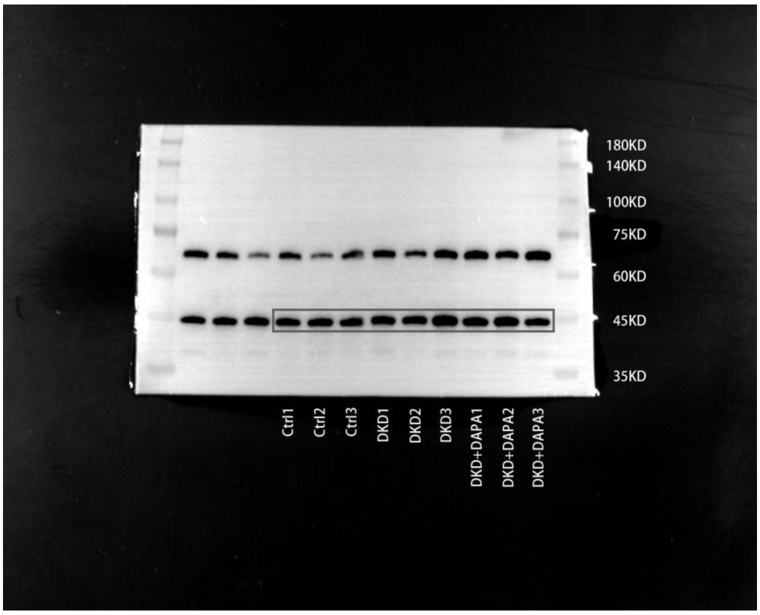

COL1

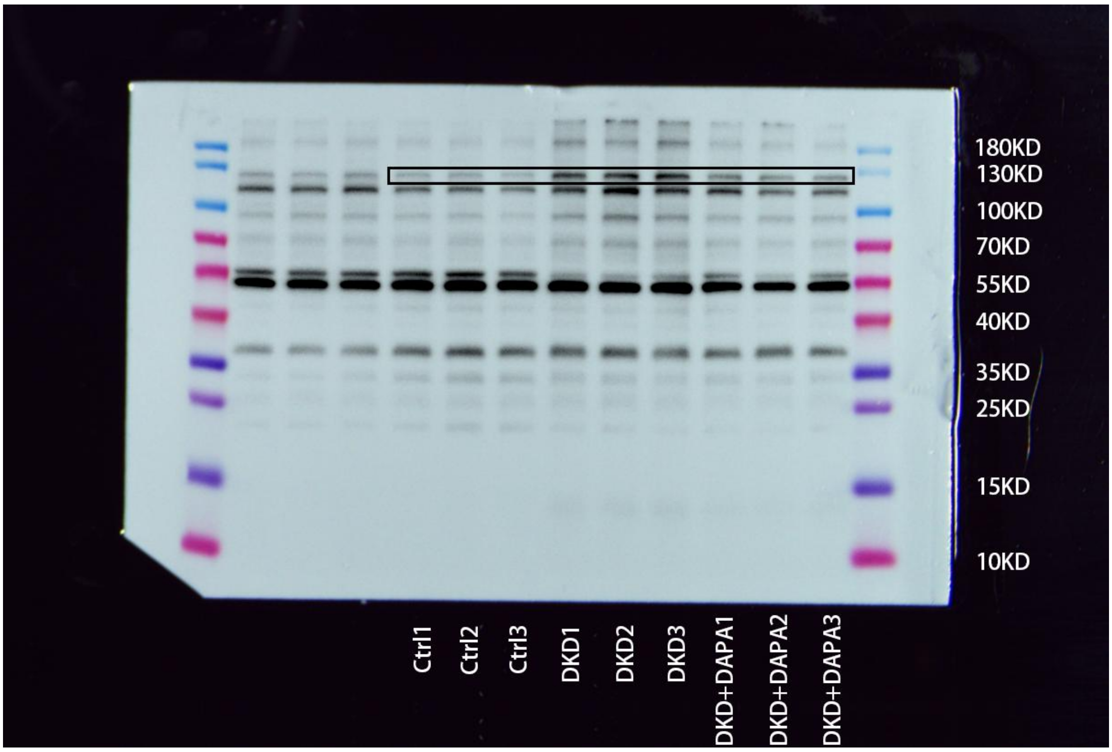

COL1 β -actin

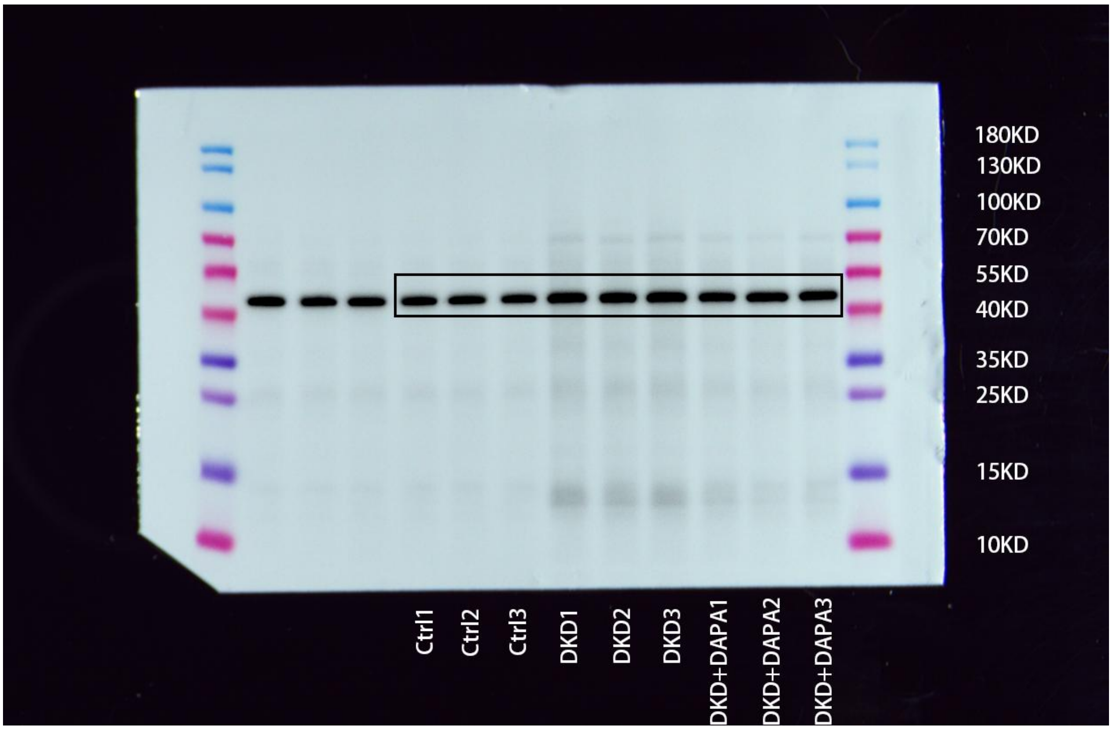

p-Smad3

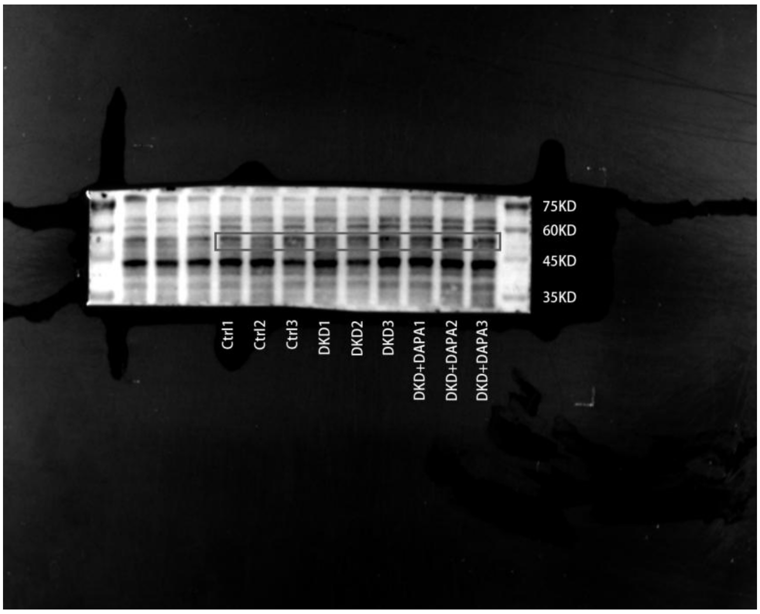

Smad3

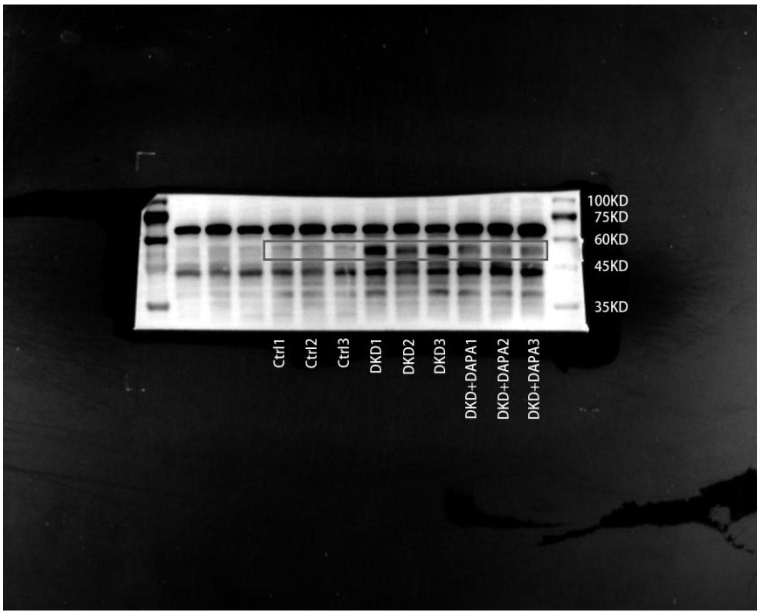

Owing to the significant interference from the stripping buffer and excessive nonspecific bands on the full membrane, P-Smad3 and Smad3 were evaluated separately on parallel gels loaded with identical amounts and sequences of samples.

Smad7

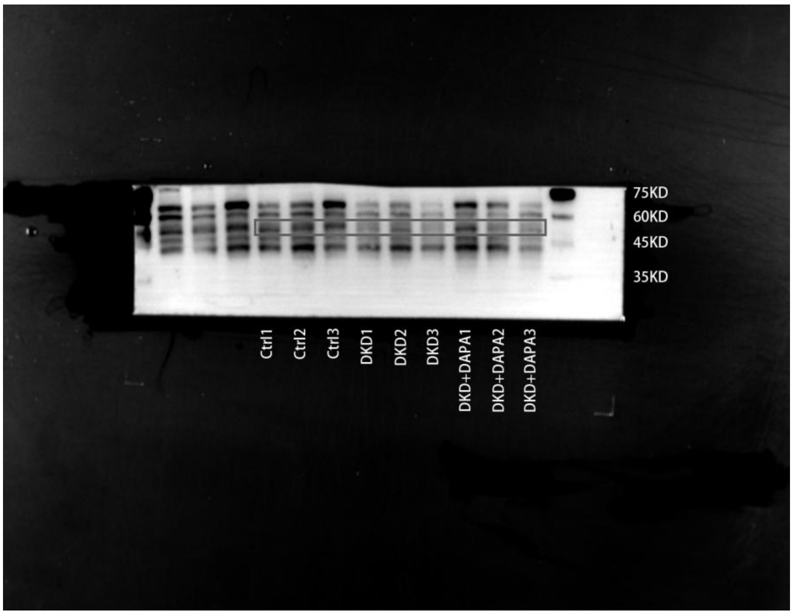

Smad7 β -actin

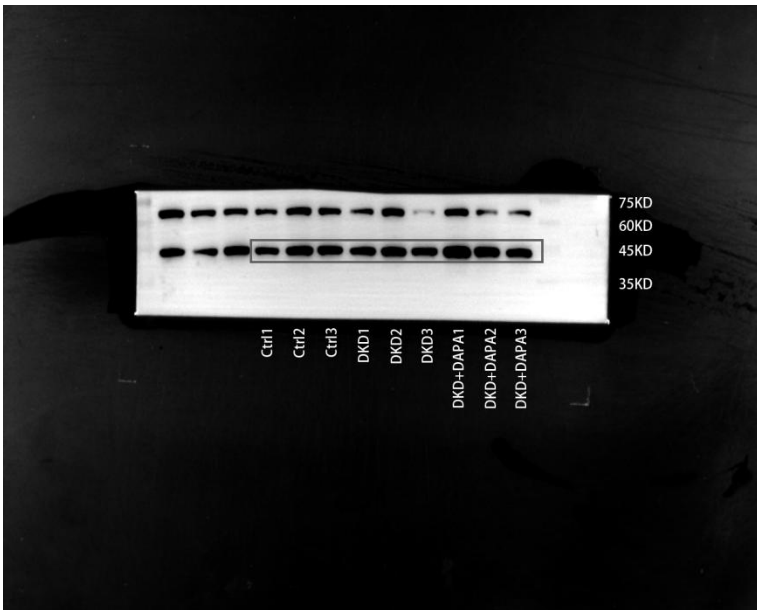

Due to excessive nonspecific bands, Smad7 was analyzed on a trimmed membrane.

TGFβ1

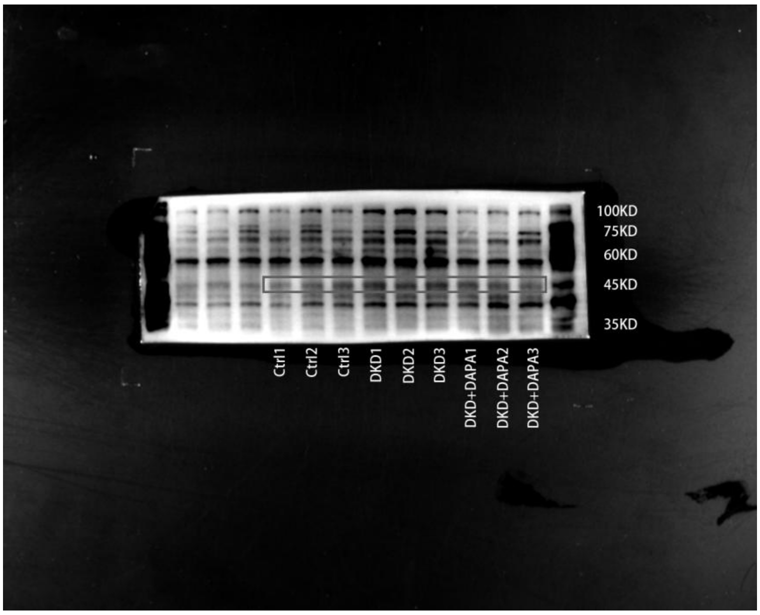

TGFβ1 β-actin

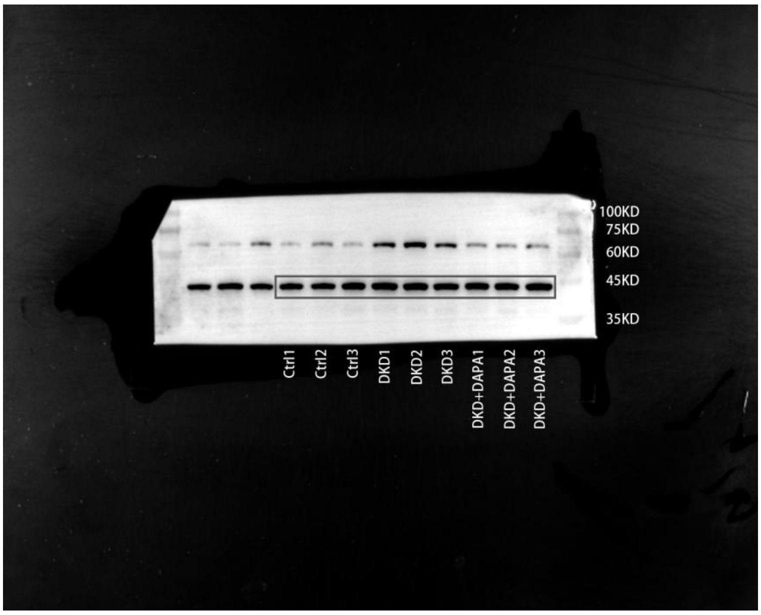

Due to excessive nonspecific bands, TGFβ1 was analyzed on a trimmed membrane.
