# Supplementary figures and images for: Dapagliflozin attenuates diabetic renal fibrosis by inhibiting macrophage-myofibroblast transition via the TGF-β1-Smad3/7 pathway
Source: PeerJ. 2026 Jun 3;14:e21321. doi: 10.7717/peerj.21321 (PMC13242187; doi:10.7717/peerj.21321)

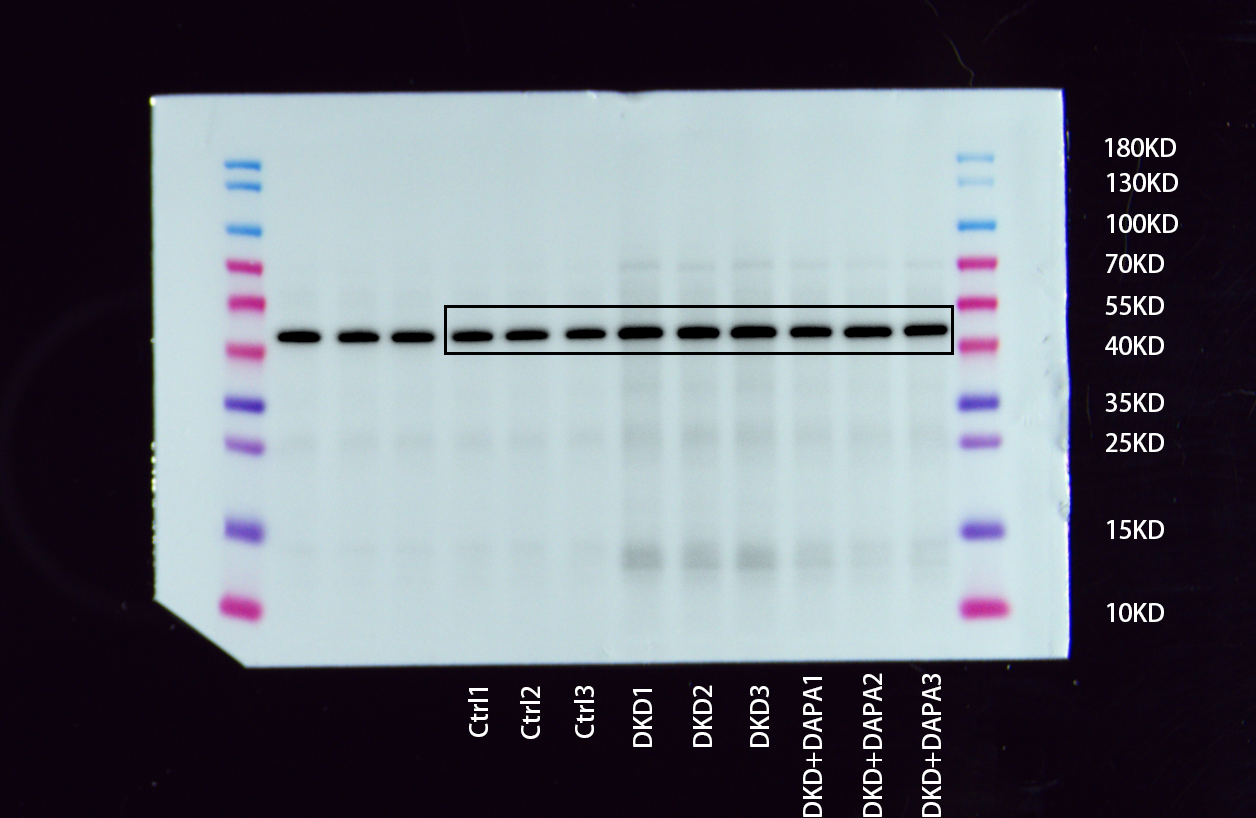

Supplement: Supplemental Information 1 [file peerj-14-21321-s001.zip › Supplementary Document/Western Blot raw data/Col-I-β-actin.png]

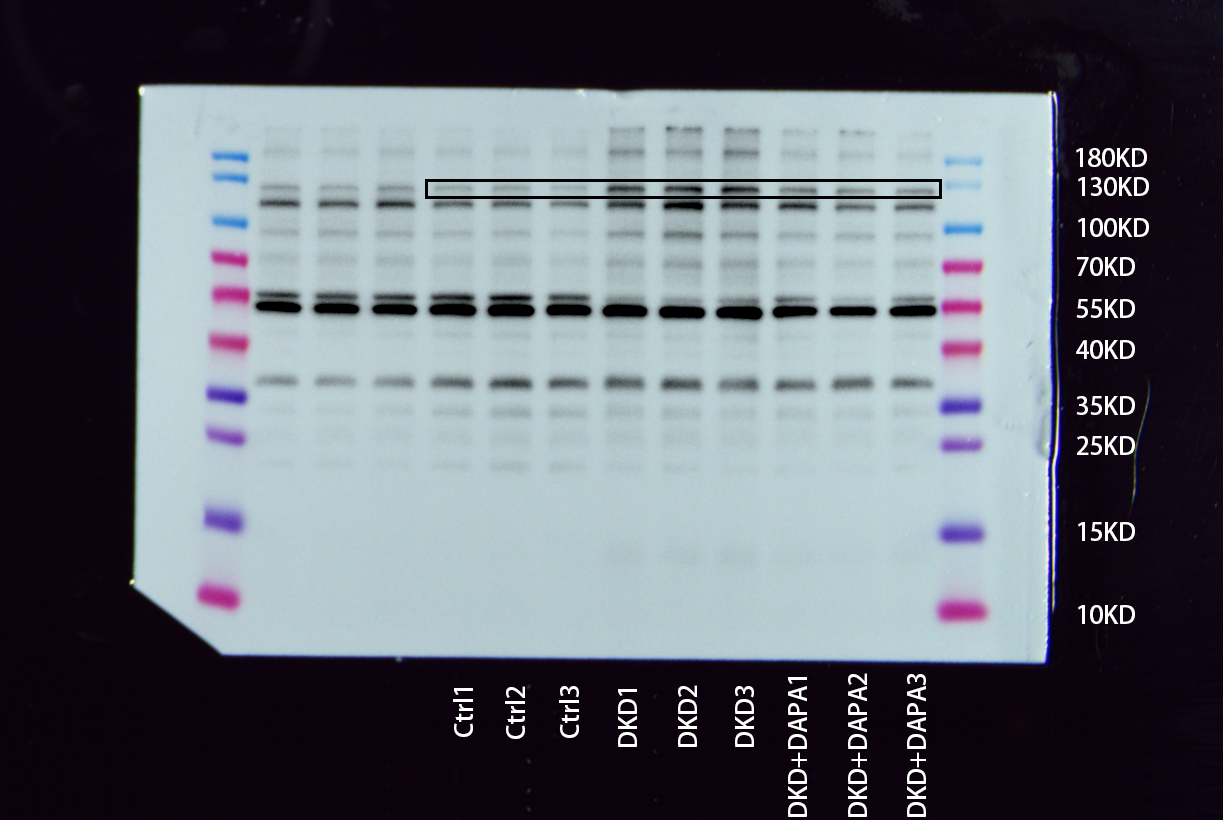

Supplement: Supplemental Information 1 [file peerj-14-21321-s001.zip › Supplementary Document/Western Blot raw data/Col-I.png]

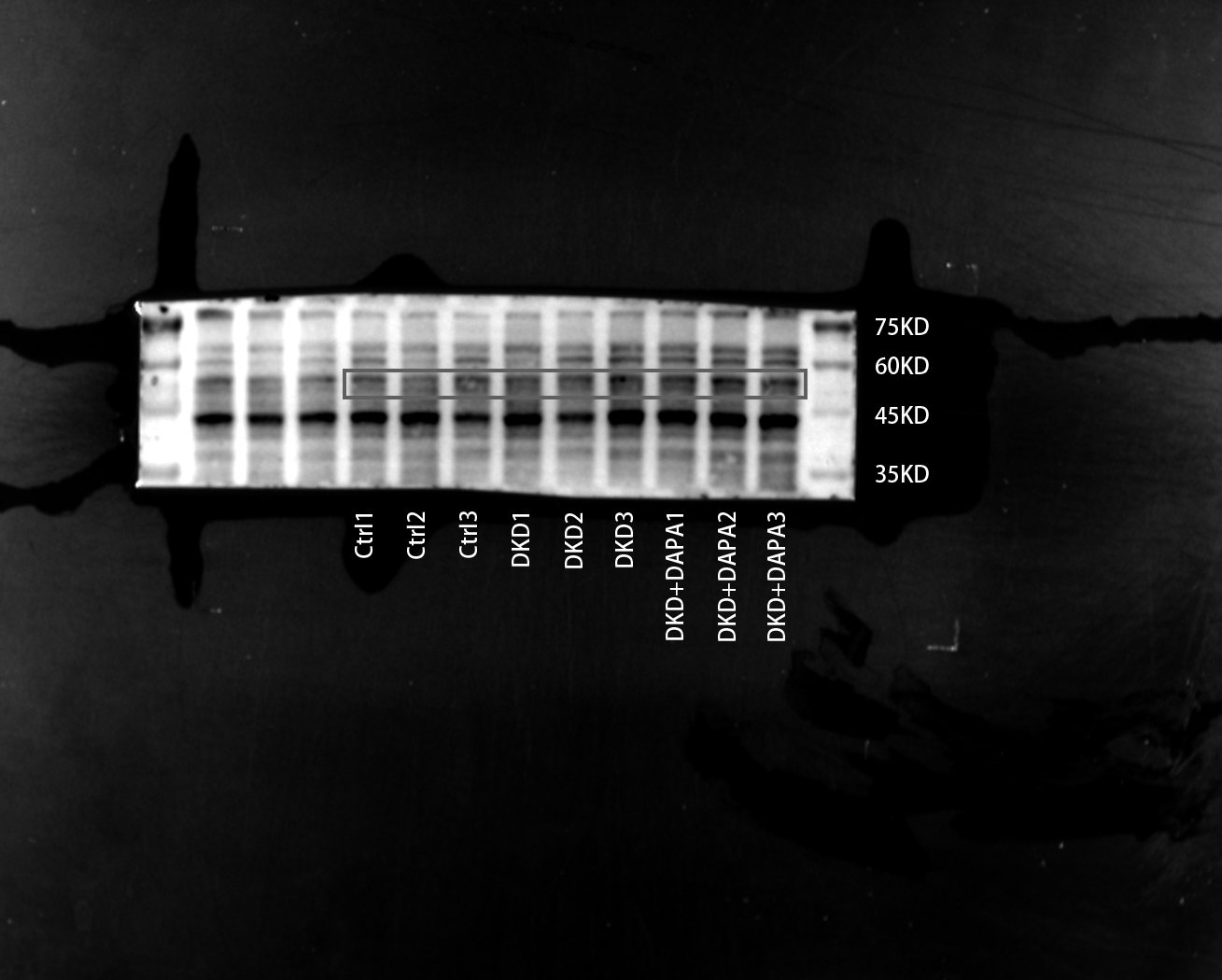

Supplement: Supplemental Information 1 [file peerj-14-21321-s001.zip › Supplementary Document/Western Blot raw data/p-Smad3 1000-5000-9.png]

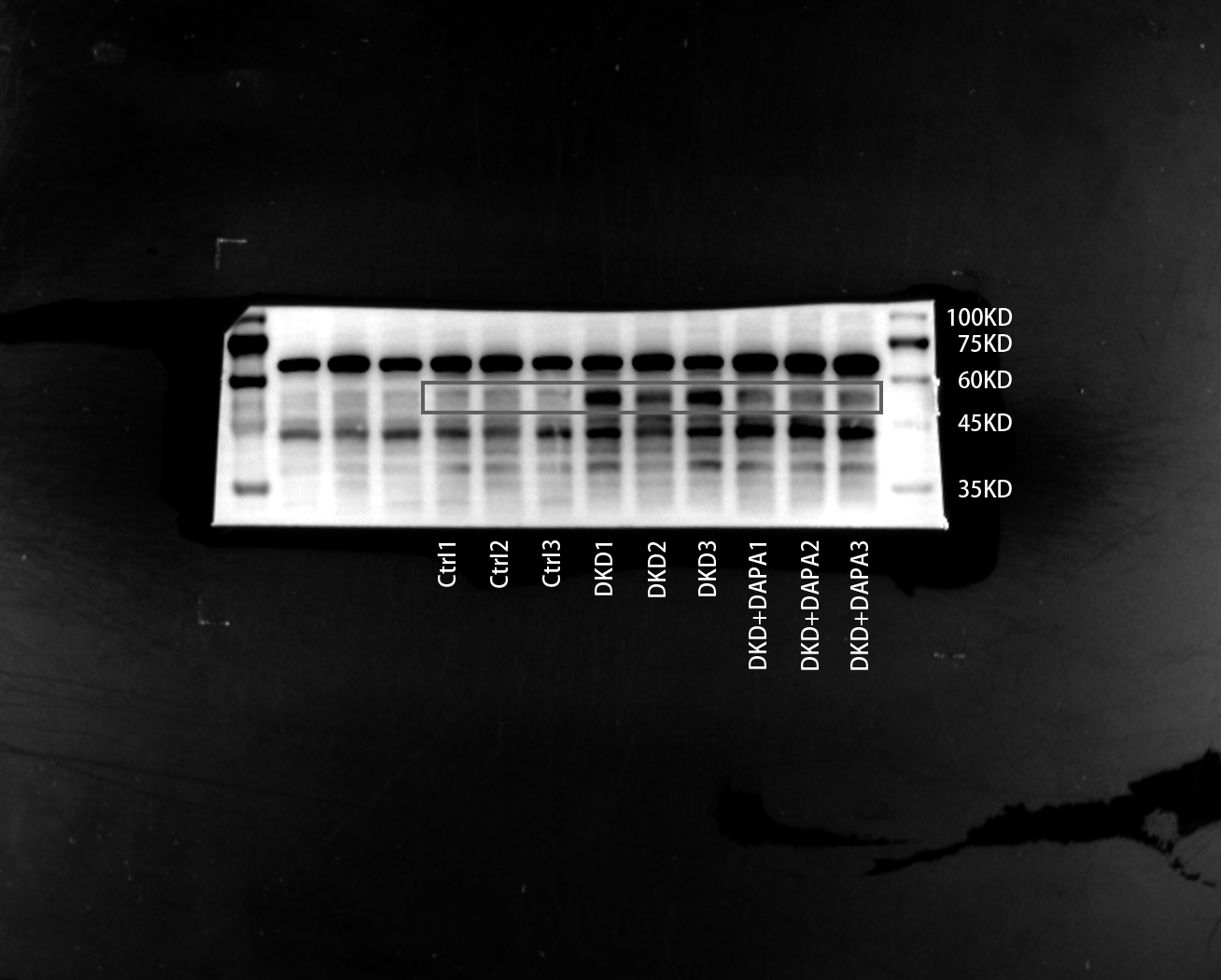

Supplement: Supplemental Information 1 [file peerj-14-21321-s001.zip › Supplementary Document/Western Blot raw data/Smad3 1000-5000-4.png]

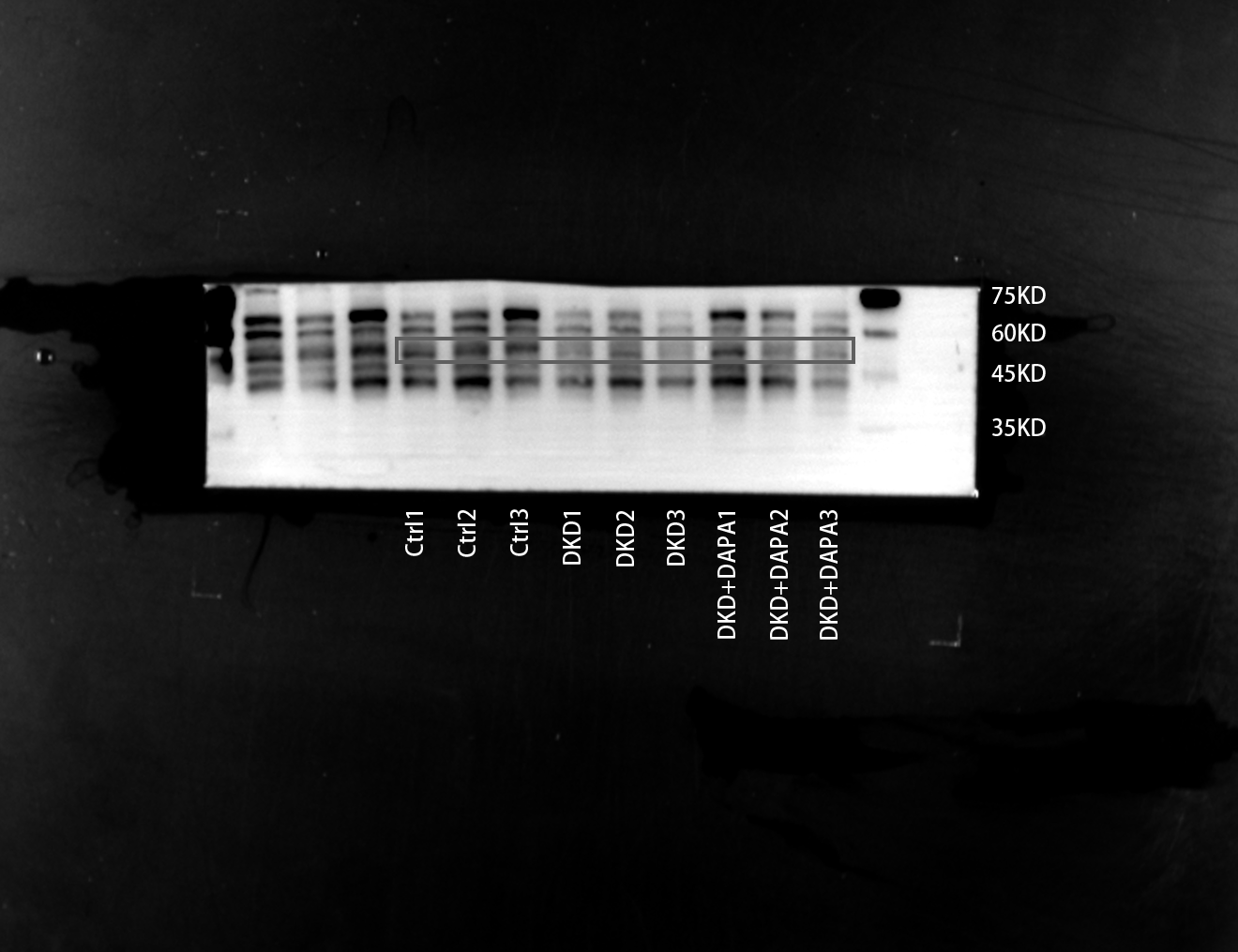

Supplement: Supplemental Information 1 [file peerj-14-21321-s001.zip › Supplementary Document/Western Blot raw data/Smad7 2000-5000-4.png]

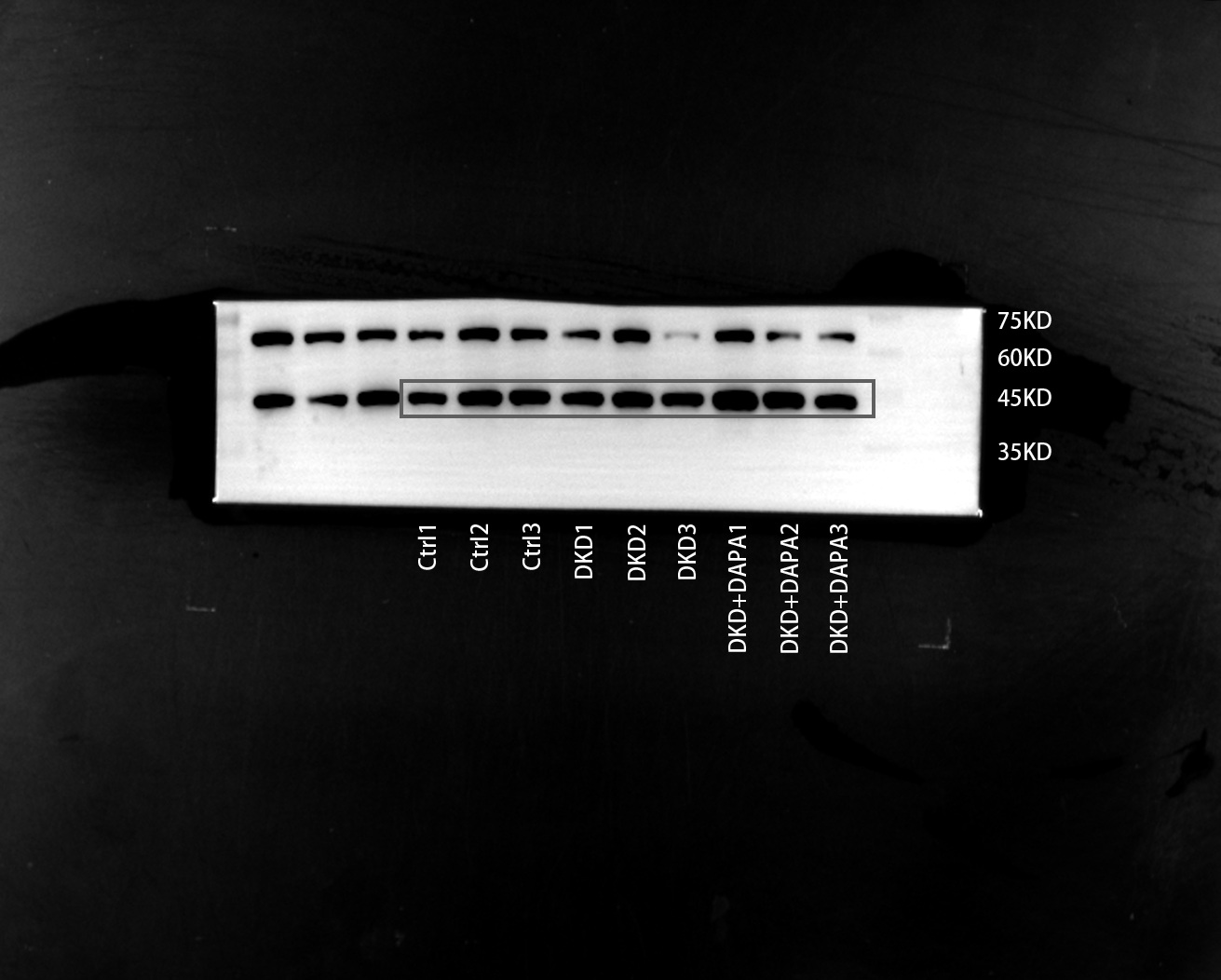

Supplement: Supplemental Information 1 [file peerj-14-21321-s001.zip › Supplementary Document/Western Blot raw data/Smad7 β-actin 1000-5000-0.png]

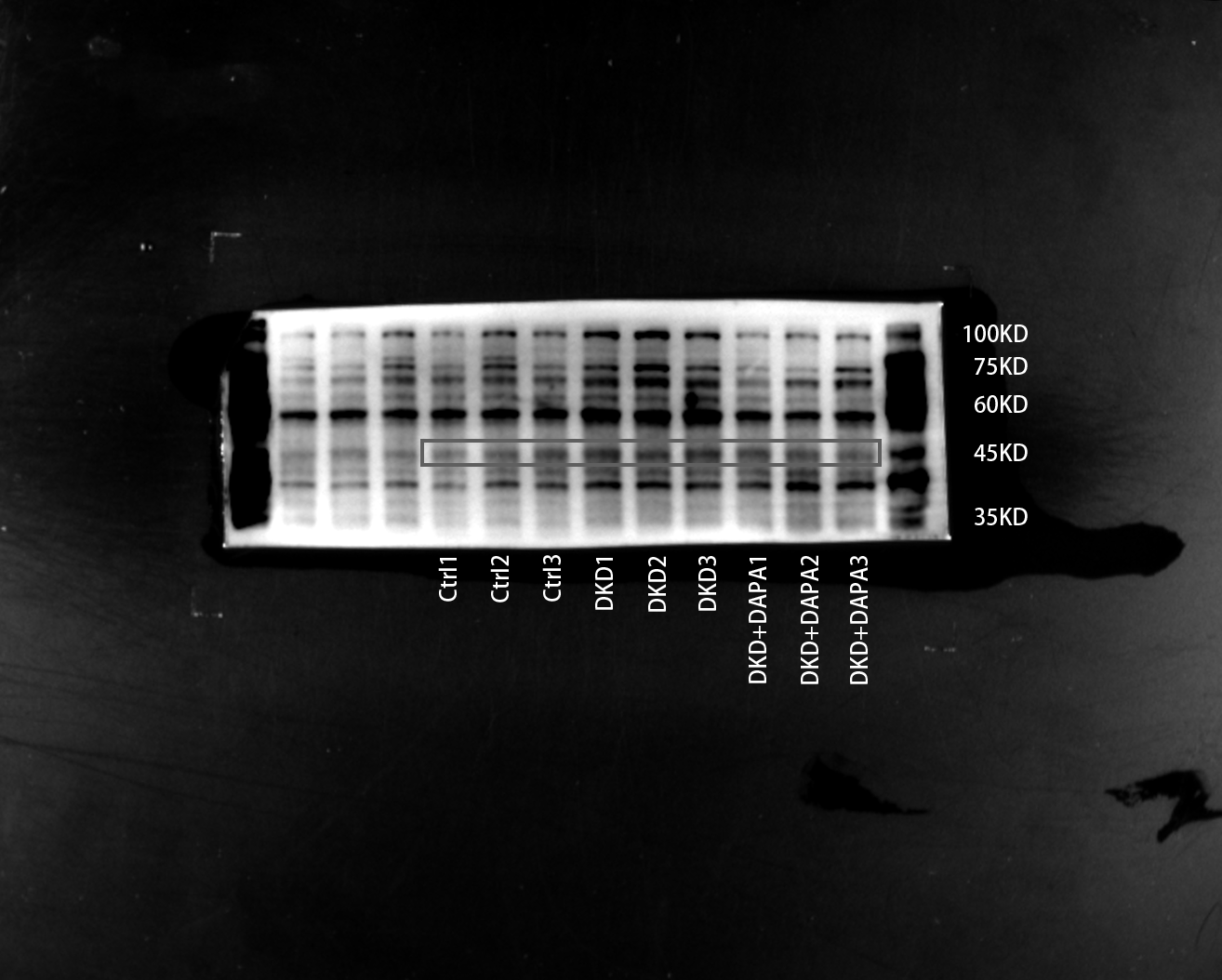

Supplement: Supplemental Information 1 [file peerj-14-21321-s001.zip › Supplementary Document/Western Blot raw data/TGFβ1 1000-5000-2.png]

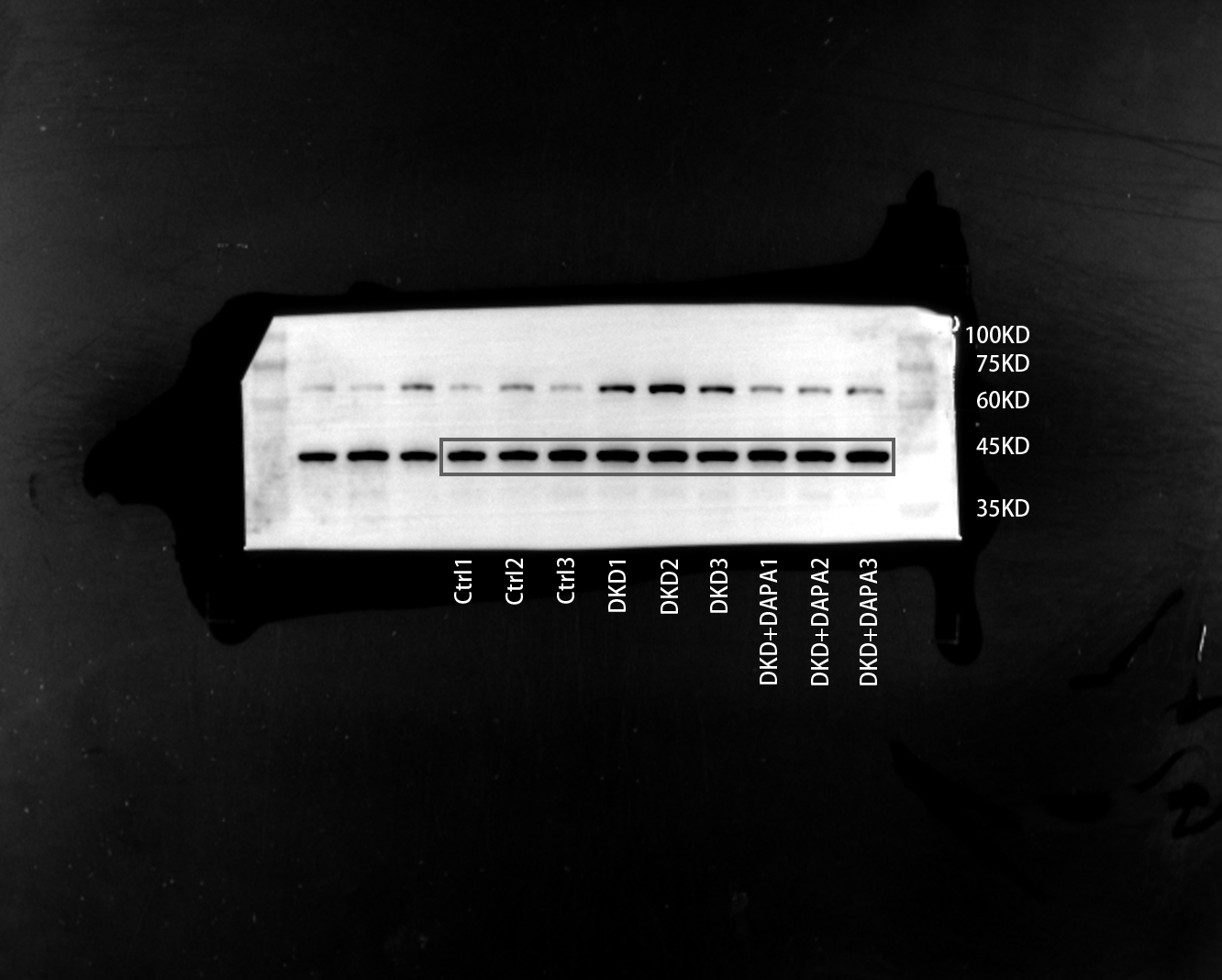

Supplement: Supplemental Information 1 [file peerj-14-21321-s001.zip › Supplementary Document/Western Blot raw data/TGFβ1 β-actin 1000-5000-0.png]

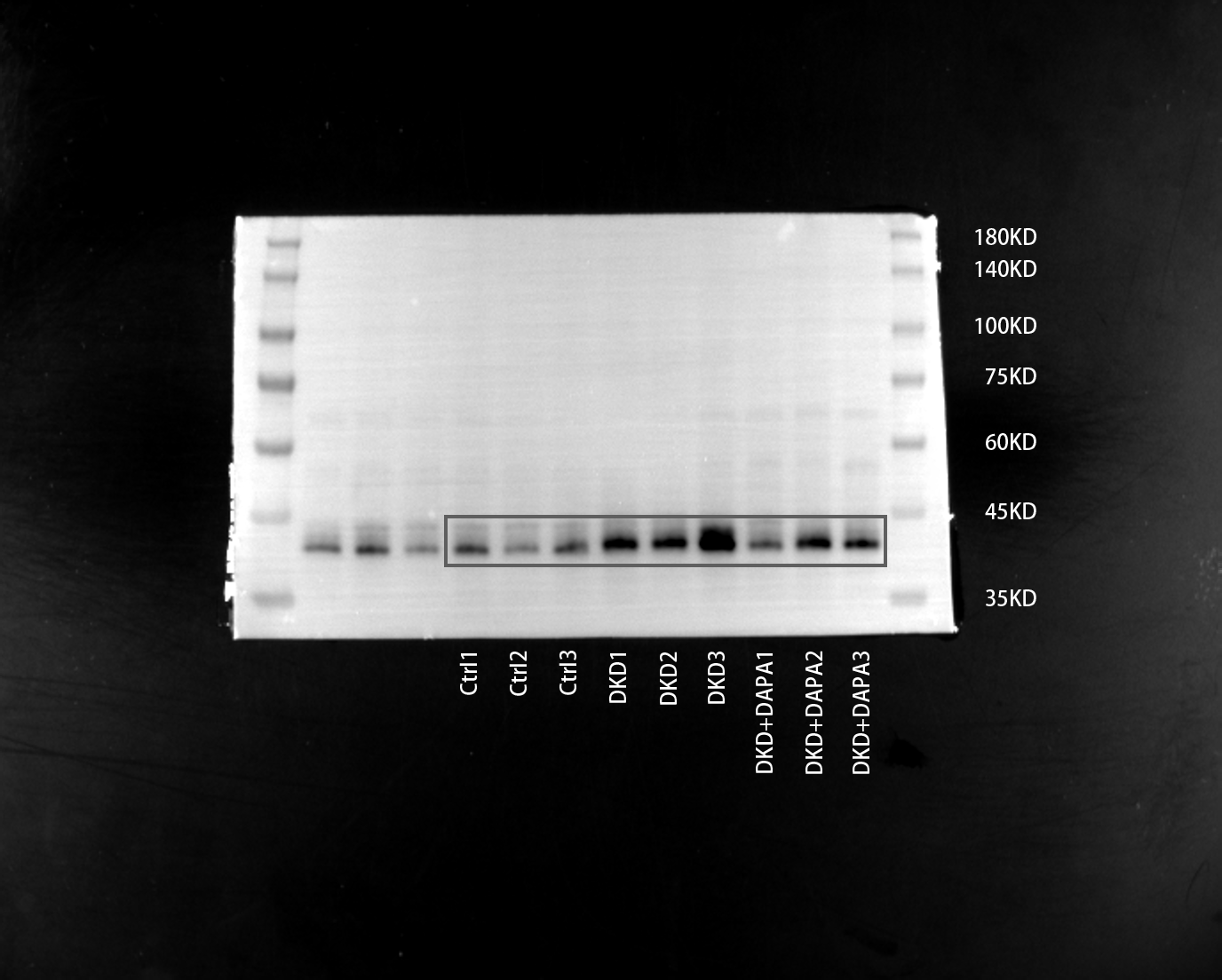

Supplement: Supplemental Information 1 [file peerj-14-21321-s001.zip › Supplementary Document/Western Blot raw data/α-SMA 1000-5000-2.png]

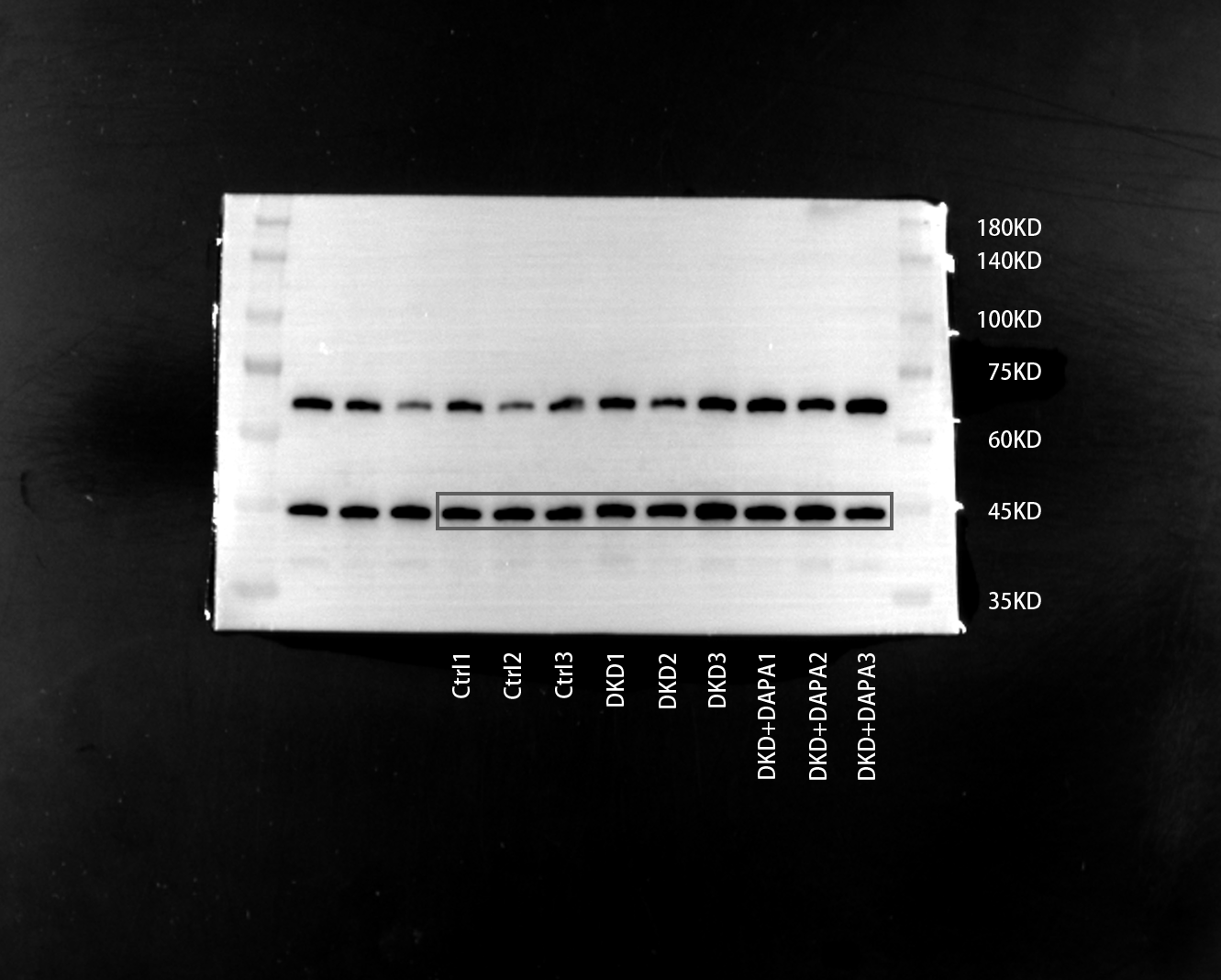

Supplement: Supplemental Information 1 [file peerj-14-21321-s001.zip › Supplementary Document/Western Blot raw data/α-SMA β-actin 1000-5000-0.png]
